# PA2G4 promotes the metastasis of hepatocellular carcinoma by stabilizing FYN mRNA in a YTHDF2-dependent manner

**DOI:** 10.1186/s13578-022-00788-5

**Published:** 2022-05-07

**Authors:** Sheng Sun, Yiyang Liu, Meiling Zhou, Jinyuan Wen, Lin Xue, Shenqi Han, Junnan Liang, Yufei Wang, Yi Wei, Jinjin Yu, Xin Long, Xiaoping Chen, Huifang Liang, Zhao Huang, Bixiang Zhang

**Affiliations:** 1 Hepatic Surgery Center, Tongji Hospital, Tongji Medical College, Huazhong University of Science and Technology, 1095 Jiefang Avenue, Wuhan, 430030 China; 2 Clinical Medical Research Center of Hepatic Surgery at Hubei Province, Wuhan, China; 3 Hubei Key Laboratory of Hepato-Pancreatic-Biliary Diseases, Tongji Hospital, Tongji Medical College, Huazhong University of Science and Technology, Wuhan, China; 4 Wuhan Institute of Biomedical Sciences, School of Medicine, Jianghan University, Wuhan, China; 5 Key Laboratory of Organ Transplantation, Ministry of Education; Key Laboratory of Organ Transplantation, National Health Commission; Key Laboratory of Organ Transplantation, Chinese Academy of Medical Sciences, Wuhan, China

**Keywords:** PA2G4, HCC, m6A, YTHDF2, FYN

## Abstract

**Background:**

Hepatocellular carcinoma (HCC) is one of the most common cancers worldwide with high mortality. Advanced stage upon diagnosis and cancer metastasis are the main reasons for the dismal prognosis of HCC in large part. The role of proliferation associated protein 2G4 (PA2G4) in tumorigenesis and cancer progression has been widely investigated in various cancers. However, whether and how PA2G4 participates in HCC metastasis is still underexplored.

**Results:**

We found that the mRNA and protein levels of PA2G4 were higher in HCC samples than in normal liver tissues, and high expression of PA2G4 in HCC was correlated with a poor prognosis, by an integrative analysis of immunohistochemistry (IHC), western blot and bioinformatic approach. Moreover, the expression of PA2G4 was elevated in HCC patients with metastases than those metastasis-free. Cell migration, invasion, phalloidin staining and western blot analyses demonstrated that PA2G4 promoted epithelial to mesenchymal transition (EMT) of HCC cells in vitro. And a lung metastasis animal model exhibited that PA2G4 enhanced metastatic ability of HCC cells in vivo. RNA-sequencing combined with dual luciferase reporter assay and evaluation of mRNA half-time indicated that PA2G4 increased FYN expression by stabilizing its mRNA transcript. Recovering the impaired FYN level induced by PA2G4 knockdown rescued the impeded cell mobilities. Furthermore, endogenous immunoprecipitation (IP) and in-situ immunofluorescence (IF) showed that YTH N6-methyladenosine RNA binding protein 2 (YTHDF2) was the endogenous binding patterner of PA2G4. In addition, RNA binding protein immunoprecipitation (RIP) and anti- N6-methyladenosine immunoprecipitation (MeRIP) assays demonstrated that FYN mRNA was N6-methyladenosine (m6A) modified and bound with PA2G4, as well as YTHDF2. Moreover, the m6A catalytic ability of YTHDF2 was found indispensable for the regulation of FYN by PA2G4. At last, the correlation of expression levels between PA2G4 and FYN in HCC tissues was verified by IHC and western blot analysis.

**Conclusions:**

These results indicate that PA2G4 plays a pro-metastatic role by increasing FYN expression through binding with YTHDF2 in HCC. PA2G4 may become a reliable prognostic marker or therapeutic target for HCC patients.

**Supplementary Information:**

The online version contains supplementary material available at 10.1186/s13578-022-00788-5.

## Background

Liver cancer is the third leading cause of cancer related death, and its incidence continues increasing [1, 2]. HCC is the predominant form of liver cancer, accounting for ~ 90% of all cases [3]. The prognosis of HCC patients has improved due to advancements in early diagnostic methods and therapeutic approaches [2]. At the same time, the prognosis remains grim and limited treatment strategies are available for HCC patients with metastases [4, 5]. However, the underlying molecular mechanism of HCC metastasis is still not fully understood.

PA2G4, also named ErbB3-binding protein 1 (EBP1), was firstly identified by Lamartine et al. as an analogue of murine p38-2G4 protein [6]. PA2G4 has two alternate isoforms, PA2G4-p42 (340 amino acids (aa)) and PA2G4-p48 (394 aa), which both regulate cellular growth and differentiation, but often have opposite effects depending on the context [7, 8]. PA2G4-p48 is the predominant form in mammalian cells, and is found both in the cytoplasm and nucleus. While PA2G4-p42 is expressed at a relative low level and is located only in the cytoplasm [7]. PA2G4 is intensively involved in tumorigenesis and cancer progression. It plays versatile roles including being a ribosomal constituent [9], transcriptional activator/repressor [10–12] and RNA/DNA/protein binding partner [13, 14] to mediate rRNA processing [15], DNA transcription [10, 11], mRNA translation [9], protein stability [16] and signal transduction [17]. The functions of PA2G4 in cancers are highly context dependent. PA2G4 was found to be highly expressed in cervical cancer, colon cancer, nasopharyngeal carcinoma and salivary adenoid cystic carcinoma [18–21], while it was found to be downregulated in HER2^+^ breast cancer [22] and bladder cancer [23]. In HCC, PA2G4 was reported to promote cell proliferation, soft agar colony generation, and tumor formation via Ebp1/p38/HIF1α signaling and MDM2-mediated downregulation of p53 [24]. However, Hu et al. reported that downregulation of PA2G4 enhanced proliferation of HCC cells [25].

N6-methyladenosin (m6A) is one of the most pervasive modifications of mRNA in eukaryotes, which exerts sophisticated regulatory effects via so-called “Writer” and “Eraser” proteins [26]. Accumulating evidence suggests that m6A regulates mRNA splicing, nuclear export, mRNA stability and translation through the recruitment of specific reader proteins or by changing the RNA structure [27, 28]. YTHDF2 was the first identified m6A reader protein that could affect the stability of mRNA [29]. Typically, YTHDF2 recognizes and binds to m6A sites in the 3’-untranslational regions (UTR) of its target mRNAs, subsequentially recruits CCR4-NOT to initiate deadenylation and decay of m6A-containing mRNAs [28]. At the same time, YTHDF2 was found to promote mRNA translation by preserving the m6A in the 5’UTR [30, 31] and increasing mRNA stability by recognizing the m6A in mRNA [32].

FYN is a non-receptor tyrosine kinases (RTK) member of the Src family kinase (SFK) [33], which participates in various of signaling transduction, such as PI3K/AKT and EGFR pathway [34–37]. In cancers, it was reported to play a pro-tumorigenic and pro-metastatic role by promoting proliferation [38, 39], enhancing migration and invasion [36, 38, 39], inhibiting apoptosis [40], inducing EMT [36] and suppressing anti-tumor immune response [33]. To date, limited literature reported the involvement of FYN in HCC.

In this study, we demonstrated that PA2G4 was highly expressed in HCC tissues compared with adjacent normal liver tissues, and elevated levels of PA2G4 in HCC were associated with a poor prognosis. PA2G4 bound to YTHDF2 and thereby stabilized the mRNA of FYN, increasing the mobility of HCC cells in vitro and promoting lung metastasis in vivo. Our results imply that PA2G4 may be a promising prognostic marker and therapeutic target for the treatment of HCC.

## Results

### PA2G4 is upregulated in HCC and high expression of PA2G4 is correlated with a poor prognosis

**Fig. 1:**
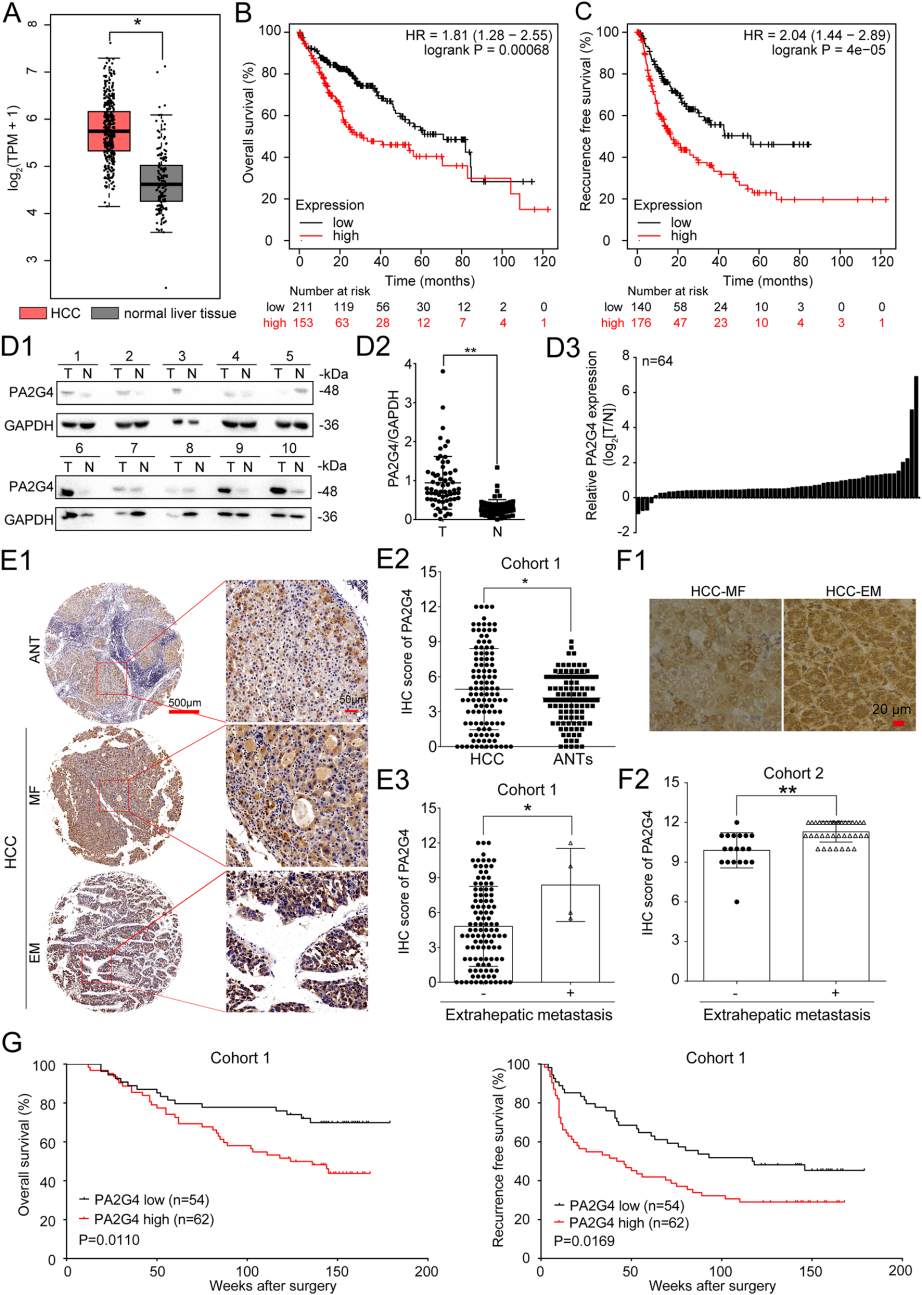
PA2G4 is upregulated in HCC and high expression of PA2G4 predicts poor prognosis. **A** Relative mRNA expression of PA2G4 in HCC (n = 369) and normal liver tissues (n = 160) analyzed by GEPIA. **B**, **C** Kaplan–Meier analyses of overall survival (**B**) and recurrence free survival (**C**) for HCC patients in TCGA database with different mRNA level of PA2G4 analyzed by Kaplan–Meier Plotter. **D** Representative western blot bands of PA2G4 in paired HCC specimens (**D1**). GAPDH as loading control. Quantification of relative PA2G4 bands intensity in HCC and adjacent non-tumorous tissues by normalizing to GAPDH (D2). Relative PA2G4 expression in HCC was shown as fold change to their respective adjacent non-tumorous liver tissues after normalizing to GAPDH (D3). **E** Representative IHC images of PA2G4 in paired HCC specimens (**E1**). HCC-MF: HCC with metastasis free; HCC-EM: HCC with extrahepatic metastases. Scale bar: red bar, 500 μm in the overview images; 50 μm in the magnified images. IHC scoring of PA2G4 in 116 pairs of HCC tissues (**E2**). IHC scoring of PA2G4 in HCC with (n = 4) or without extrahepatic metastasis (n = 112) (**E3**). **F** Representative images of IHC analysis for PA2G4 in HCC with (n = 38) or without (n = 19) extrahepatic metastasis in the following-up period (**F1**). Scale bar, red bar, 20 μm. IHC scoring of PA2G4 in each group (**F2**). **G** Kaplan–Meier analyses of overall survival time and recurrence-free survival in HCC patients with different protein expression of PA2G4. Data was shown as Mean ± SD. Two-tailed Student t test for (**A**, **D2**, **E2**, **E3** and **F2**); Log rank test for (**B**, **C** and **G**). *p < 0.05, **p < 0.01. TPM: transcripts per million; HCC: hepatocellular carcinoma; T: liver tumor; N: adjacent non-tumorous liver tissue; IHC: immunohistochemistry

To explore the clinical relevance of PA2G4 for HCC progression, we firstly evaluated its mRNA expression pattern in TCGA database. The mRNA levels of PA2G4 were significantly higher in HCC specimens than in normal liver tissues (Fig. 1A). In addition, HCC patients with higher PA2G4 mRNA expression displayed shorter overall survival time (OS), recurrence-free survival time (RFS), progression-free survival time (PFS) and disease-free survival time (DFS) (Fig. 1B, C and Additional file 1: Fig. S1 A, 1B). Western blot analysis was performed on 64 pairs of HCC specimens, and PA2G4-p48 was identified as the main expressed isoform, with the PA2G4-p42 isoform almost undetectable (Fig. 1D1). At the same time, the expression of PA2G4 was significantly higher in HCC tissues than in corresponding adjacent normal tissues (ANTs) (Fig. 1D2 and 1D3). IHC analysis of 116 paired HCC specimens (cohort 1) further validated the increased PA2G4 levels in HCC tissues compared with matched adjacent normal tissues (Fig. 1E1, 1E2, Additional file 1: Fig. S1D and Table S1). Moreover, the average IHC score of PA2G4 in HCC patients with extrahepatic metastases was significantly higher than those without metastasis (Fig. 1E1 and 1E3). IHC analysis in HCC patient cohort 2 showed that the expression of PA2G4 was remarkably elevated in HCC specimens diagnosed with extrahepatic metastases in the following-up than those metastasis-free (Fig. 1F). Additionally, Chi-squared test indicated that higher expression of PA2G4 in HCC was associated with advanced Barcelona Clinic Liver Cancer (BCLC) stages (Additional file 1: Table S2). Kaplan–Meier analysis indicated that HCC patients with higher expression of PA2G4 exhibited worse overall survival and higher tumor recurrence rates (Fig. 1G). Moreover, univariate Cox regression analysis showed that higher expression of PA2G4 predicted shorter OS and RFS time, and multivariate Cox regression analysis demonstrated that PA2G4 was an independent risk factor for recurrence, though not for survival (Additional file 1: Table S3).

### Upregulating PA2G4 induces partial EMT in HCC cells in vitro and promotes lung metastasis in vivo

**Fig. 2:**
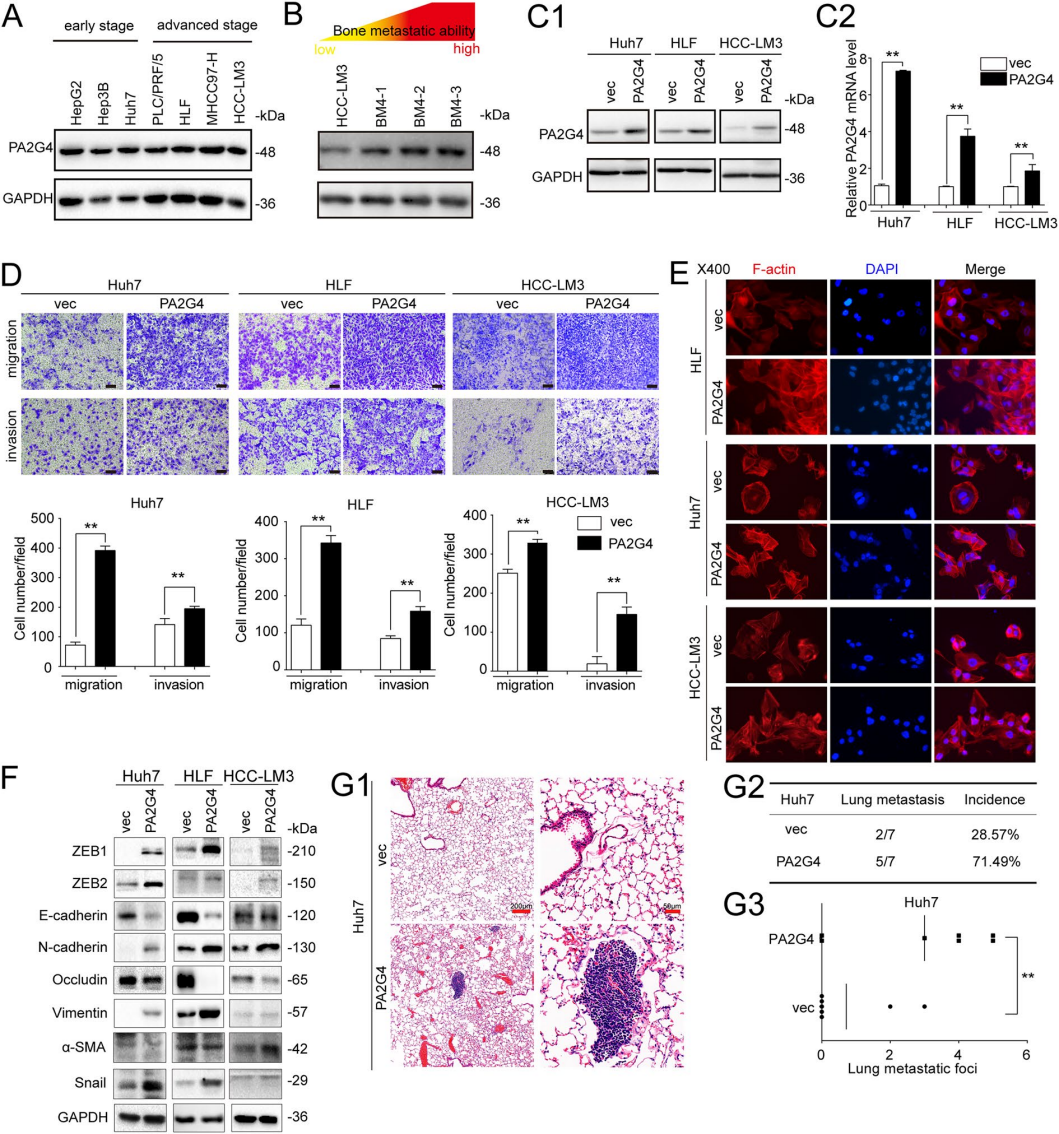
Upregulating PA2G4 induces partial EMT of HCC cell in vitro and promotes lung metastasis in vivo. **A** Western blot analyses of endogenous PA2G4 protein level in primary HCC cell lines. **B** Western blot analyses for PA2G4 expression in HCC cell lines with elevated bone metastatic abilities. BM: bone metastasis. **C** PA2G4 overexpression efficacy in the indicated cell lines were evaluated by western blot (**C1**) and qRT-PCR (**C2**). Data normalized to GAPDH and are shown as the fold change to their respective control cells. **D** Representative images of migration and invasion assay in the PA2G4 overexpressing HCC cells (upper panel). Scale bar: black bar, 100 μm. Quantification of cells migrated and invaded (lower panel). **E** Phalloidin staining (red) for F-actin in PA2G4 overexpressing HCC cells. DAPI (blue) for nuclei. Magnification, 400-fold. **F** Western blot analysis of the indicated epithelial to mesenchymal transition related protein markers in PA2G4 overexpressing cells. **G** Lung metastases formation after tail-vein injection of Huh7/PA2G4 cells. H&E staining for lung metastatic foci (**G1**); Lung metastatic incidence (**G2**) and metastatic numbers (**G3**) in mice bearing Huh7/vec or Huh7/PA2G4 cells were calculated. Scale bar: red bar, 200 μm in the overview images; 50 μm in the magnified images. GAPDH as loading control in (**A**, **B**, **C1** and **F**). Data was shown as Mean ± SD. Two-tailed Student t test for (**C2**, **D** and **G3**). **p < 0.01. vec: vector

Western blot analysis showed that the endogenous expression of PA2G4 in primary HCC cell lines was comparable, with PA2G4-p48 as the main expressed protein isoform (Fig. 2A). While the expression levels of PA2G4 in HCC cells with the same genetic background were in accordance with metastatic ability [41], which further implied its involvement in HCC metastasis process (Fig. 2B). We then selected Huh7, HLF and HCC-LM3 cells, which represent HCC in different grades (Additional file 1: Table S4) for further investigation. PA2G4 was then stably overexpressed in the HCC cells lines by lentivirus (Huh7/PA2G4, HLF/PA2G4 and HCC-LM3/PA2G4), western blot analysis and qRT-PCR were performed to examined the overexpression efficacy (Fig. 2C). Transwell assays demonstrated that overexpressing PA2G4 augmented the migration and invasion abilities of HCC cells (Fig. 2D). Phalloidin staining for actin fibers (F-actin) showed that PA2G4 induced actin cytoskeleton reorganization (Fig. 2E). At the same time, western blot analysis showed that the expression of mesenchymal-like protein markers (N-cadherin, ZEB1, ZEB2, Vimentin and Snail) were higher in PA2G4 overexpressing cells than in their respective control cells, while the level of epithelial markers (E-cadherin, Occludin and α-SMA) decreased after upregulating PA2G4 expression (Fig. 2F). Overall, upregulating PA2G4 in HCC cell lines induced partial EMT and increased their mobility in vitro.

We then used Huh7/PA2G4 and corresponding control cells to establish a lung metastatic animal model by tail-vein injection in nude mice. After 60 days of inoculation, the mice were sacrificed for H&E staining of lungs to identify metastatic foci (Fig. 2G1). The incidence of lung metastasis in the mice inoculated with Huh7/PA2G4 cells was higher than in those inoculated with corresponding control cells (Fig. 2G2). At the same time, the numbers of metastatic foci in the Huh7/PA2G4 group were higher than in the control groups with statistically significance (Fig. 2G3).

### Downregulating PA2G4 inhibits the mobility of HCC cells in vitro and lung metastasis in vivo

**Fig. 3:**
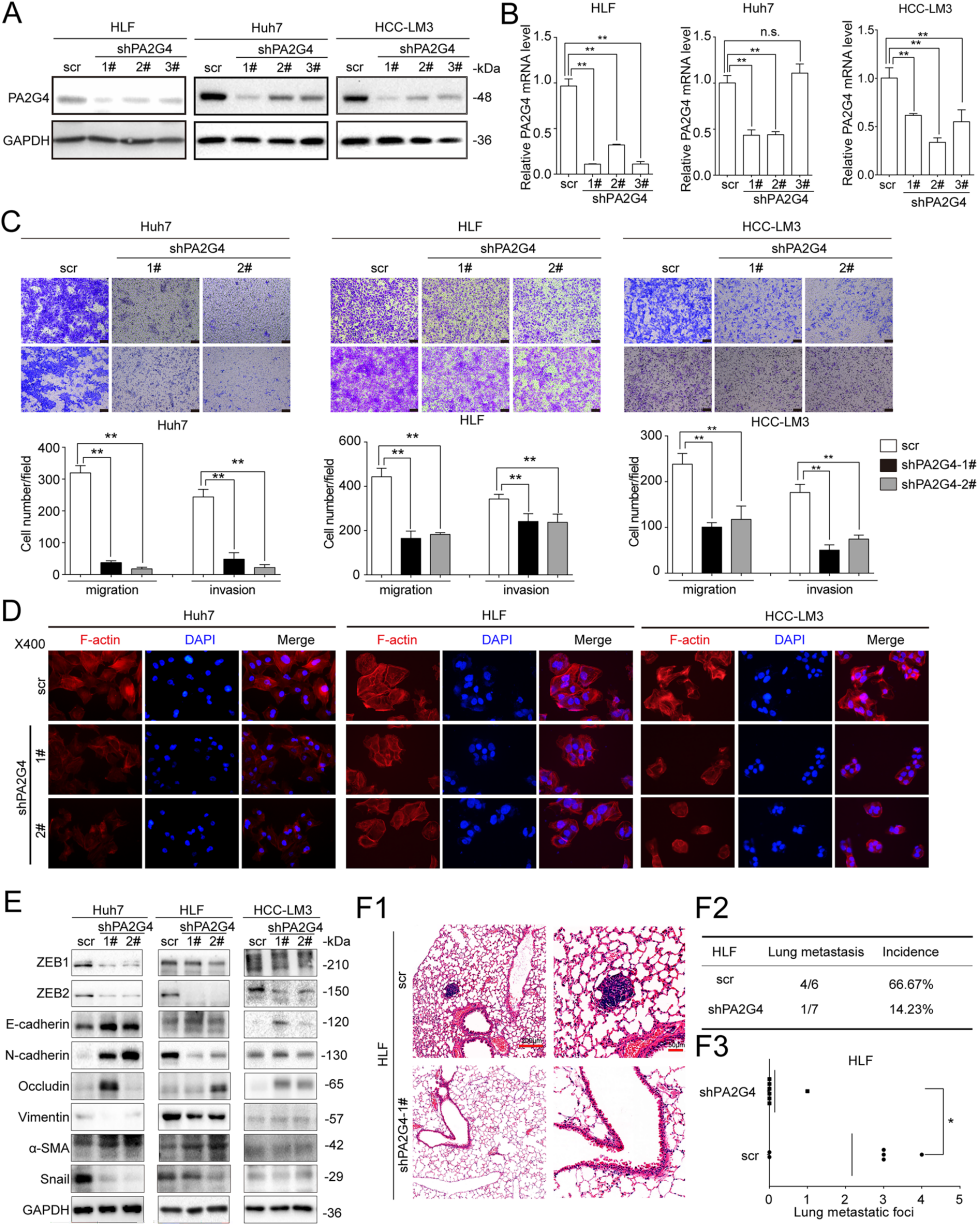
Downregulating PA2G4 inhibits mobilities of HCC cells in vitro and lung metastasis in vivo. **A**, **B** Western blot (**A**) and qRT-PCR (**B**) analysis of PA2G4 knocking down efficacy in the indicated cell lines. Data normalized to GAPDH and are shown as the fold change to their respective control cells. **C** Representative images of migration and invasion after knocking down PA2G4 expression in the indicated cells (upper panel). Scale bar, black bar, 100 μm. Quantification of cells migrated and invaded in each group (lower panel). **D** Phalloidin staining for F-actin (red) in the indicated HCC cells with PA2G4 knocked down. DAPI (blue) for nuclei. Magnification, 400-fold. **E** Western blot analyses of the indicated protein level after knocking down PA2G4. GAPDH as loading control. **F** Lung metastases in mice bearing HLF/shPA2G4-1# and their control cells. H&E staining of lung metastatic foci (**F1**); Lung metastasis incidence (**F2**) and metastatic numbers (**F3**) were calculated. Scale bar: red bar, 200 μm in the overview images; 50 μm in the magnified images. Data was shown as Mean ± SD. Two-tailed Student t test for (**B**, **C** and **F3**). *p < 0.05, **p < 0.01. scr: scramble; sh: small inference RNA. n.s.: no significance

The expression of PA2G4 was stably knocked down in the same HCC cell lines (Huh7/shPA2G4, HLF/shPA2G4 and HCC-LM3/shPA2G4) (Fig. 3A and B). The transwell assay indicated that knocking down PA2G4 in HCC cells impaired their migration and invasion ability (Fig. 3C). Phalloidin staining revealed that HCC cells with lower PA2G4 expression had a decreased rearrangement of F-actin compared with their respective control cells (Fig. 3D). In addition, western blot assay showed that downregulating PA2G4 increased the expression of E-cadherin and α-SMA, while decreased the level of ZEB1, ZEB2 and Snail (Fig. 3E).

HLF/shPA2G4-1# and corresponding control cells were then used to establish the lung metastatic animal model as described above, and the incidence of lung metastasis was lower in mice bearing HLF/shPA2G4 cells than in those inoculated with control cells (Fig. 3F1 and F2). At the same time, the number of micro-metastatic foci in mice injected with HLF/shPA2G4 cells was significantly lower than in those injected with control cells (Fig. 3F3).

### FYN is a downstream effector of PA2G4 in HCC

**Fig. 4:**
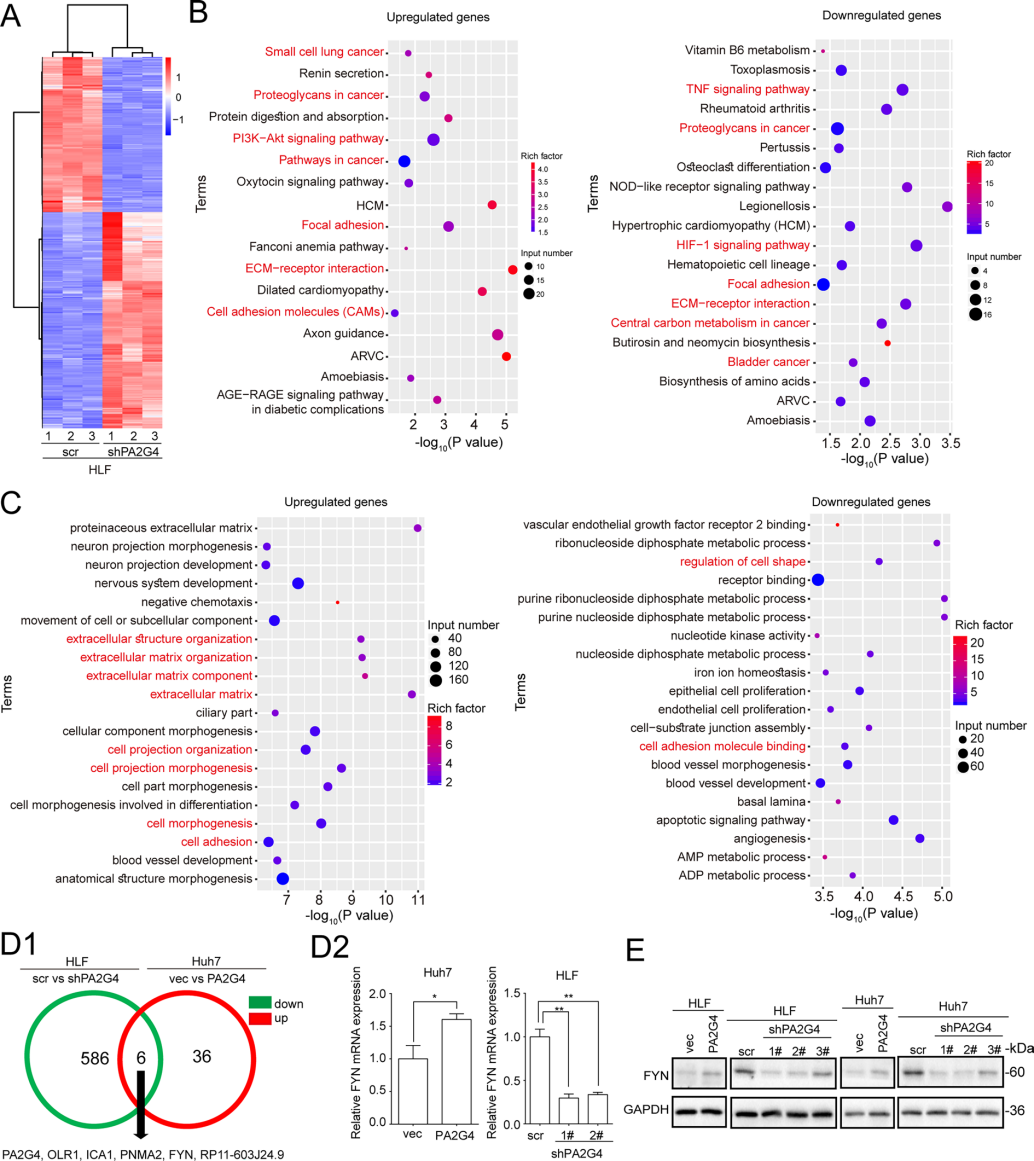
FYN is a downstream effector of PA2G4 in HCC. **A** Heatmap of differentially expressed genes between HLF/shPA2G4 and its control cells. Triplicates were used for RNA-Seq in each group. **B**, **C** KEGG (**B**) and GO (**C**) analyses for the upregulated and downregulated genes after knocking down PA2G4 in HLF cells. Red color was used to highlight the cancer related or cell mobility related terms. **D** HLF cells with PA2G4 knocked down and Huh7 cells with PA2G4 overexpressed were applied to found out the positive regulated downstream effectors of PA2G4 (log2(FC) ≥ 1 and p value < 0.05). Venn diagram for overlapping the downregulated genes in HLF/shPA2G4 cells (green circle) and the upregulated genes in Huh7/PA2G4 cells (red circle), compared with their respective control cells (**D1**). qRT-PCR of FYN expression in the indicated cells (**D2**). Data normalized to GAPDH and are shown as the fold change to their respective control cells. **E** Western blot analyses of FYN after manipulating PA2G4 level in Huh7 and HLF cells. GAPDH as loading control. Data was shown as Mean ± SD. Two-tailed Student t test for (**D2**); Overrepresentation analysis test for (**B** and **C**). *p < 0.05, **p < 0.01

Next, RNA-Seq was performed in triplicate HLF/shPA2G4-1# and control cells to reveal the landscape of the downstream targets of PA2G4. A total of 1444 genes were found to be differentially expressed after knocking down PA2G4 in HLF cells (log_2_(FC) ≥ 1 and p value < 0.05), among which 852 were up- and 592 downregulated (Fig. 4A). Kyoto Encyclopedia of Genes and Genomes (KEGG) analyses indicated that the differentially expressed genes between HLF/shPA2G4-1# and the control cells were involved in many signaling pathways which are crucial for cancer progression (Fig. 4B). Notably, gene signatures such as focal adhesion, ECM-receptor interaction, and cell adhesion molecules were enriched, implying the involvement of PA2G4 in cell mobility (Fig. 4B). Gene ontology (GO) analyses demonstrated that PA2G4-regulated genes were enriched in gene clusters such as extracellular organization, cell adhesion and cell shape, which are related to cell mobility and cancer metastasis (Fig. 4C). Taken together, these results revealed a critical role of PA2G4 in cancer progression, and especially metastasis.

To further identify the conserved downstream effectors of PA2G4, RNA-Seq was performed in Huh7/PA2G4 and corresponding control cells. A total of 73 genes were found to be differentially expressed after overexpressing PA2G4 in Huh7 cells (log2(FC) ≥ 1 and p value < 0.05), among which 42/31 were up-/down-regulated (Additional file 1: Fig. S2A). Given that PA2G4 was proven to play a pro-metastatic role in HCC cells, we performed a Venn diagram analysis of the decreased genes in HLF/shPA2G4 cells with the increased genes in Huh7/PA2G4 cells, to identify the genes that are positively regulated by PA2G4. Except for PA2G4, four additional protein coding genes (OLR1, ICA1, PNMA2, FYN) were finally obtained (Fig. 4D1), and qRT-PCR confirmed that they were positively regulated by PA2G4 in HLF/shPA2G4 and Huh7/PA2G4 cells (Fig. 4D2 and Additional file 1: Fig. S2B). Among these genes, we noticed that FYN, a proto-oncogene and a Src family tyrosine kinase, was reported to involved in EMT and to promoted cancer metastasis in colon cancer, gastric cancer, and pancreatic cancer [36, 39, 40]. Further qRT-PCR and western blot analyses demonstrated that PA2G4 promoted the mRNA and protein expression of FYN in HCC cell lines (Fig. 4E, Additional file 1: Fig. S2C–2F).

### YTHDF2 is an endogenous binding partner of PA2G4

**Fig. 5:**
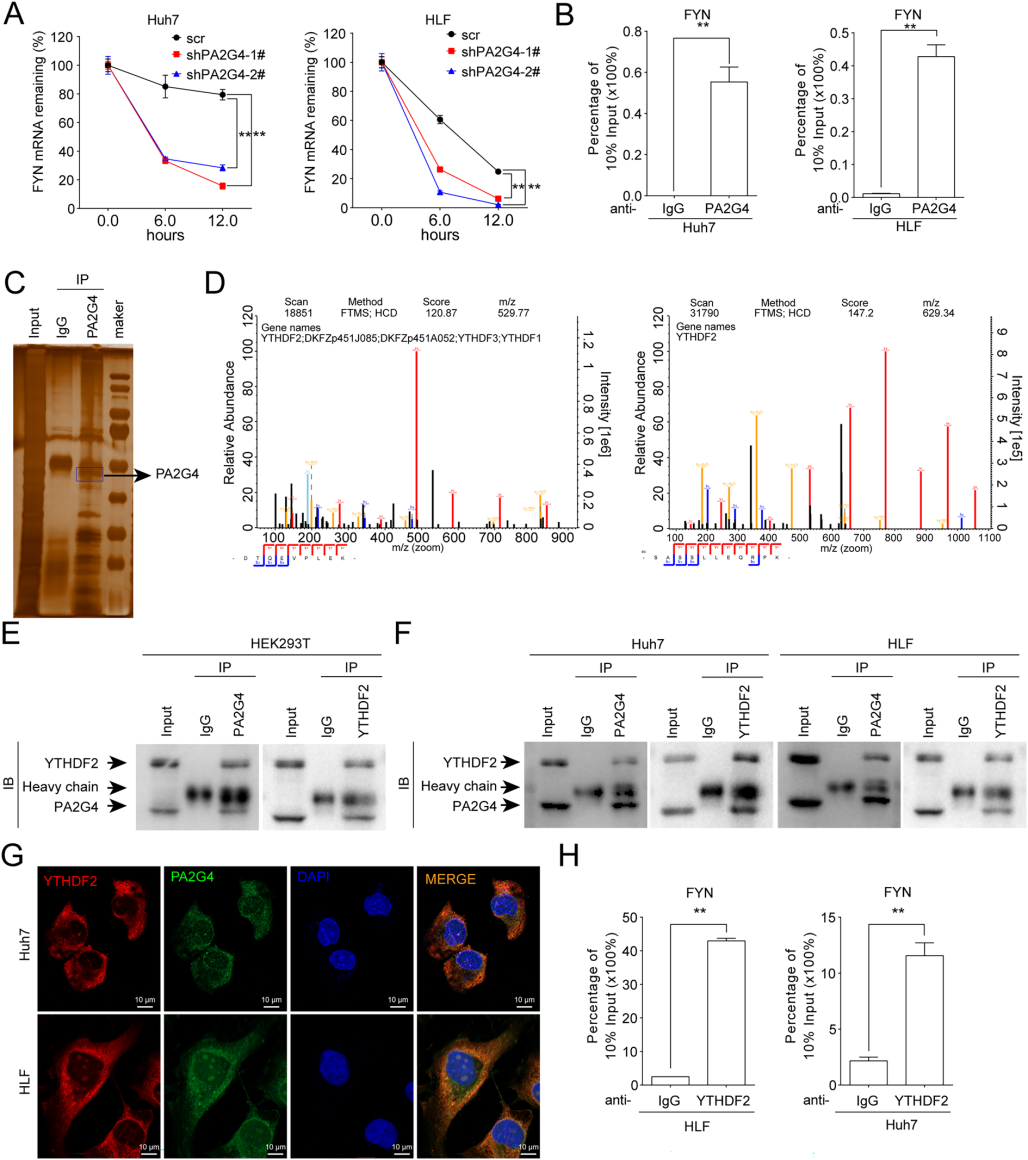
YTHDF2 is an endogenous binding patterner of PA2G4. **A** qRT-PCR of the relative FYN mRNA expression after treating with actinomycin D for the indicated time in PA2G4 knocked down cells. Data normalized to HPRT1 and are shown as percentage of the relative level in 0 h. **B** RIP assay for the binding of FYN mRNA with PA2G4 in the indicated cells. Data was shown as percentage of FYN mRNA in the anti-PA2G4 precipitates against 10% input. **C** HEK-293T cells were transduced with pcDNA3.1-PA2G4 plasmids to overexpress PA2G4, and then subjected to anti-PA2G4 immunoprecipitation. Sliver staining of the protein enriched by anti-PA2G4 antibody in HEK-293T cells. Black arrow pointed to PA2G4 bands. **D** The peptide spectrum of YTHDF2 by mass spectrum assay. **E**, **F** The binding between PA2G4 and YTHDF2 were detected by endogenous immunoprecipitation in HEK-293T (**E**) and in HCC cells (**F**). **G** Representative confocal images of the co-localization of YTHDF2 and PA2G4. Red, YTHDF2; green, PA2G4; Blue, DAPI. Scale bar, white bar, 10  µM. **H** RIP assay for the binding of FYN mRNA with YTHDF2 in the indicated cells. Data was shown as percentage of FYN mRNA in the anti-YTHDF2 precipitates against 10% input. Data was shown as Mean ± SD. Two-tailed Student t test for (**A**, **B** and **H**). **p < 0.01. IP: immunoprecipitation; IB: immunoblotting

Given that the mRNA of FYN was positively regulated by PA2G4, a dual luciferase reporter assay was performed to explore whether PA2G4 increased FYN expression by enhancing its transcription. The results showed that knocking down PA2G4 did not affect the transcription activity of FYN (Additional file 1: Fig. S3A). At the same time the half-life of FYN mRNA following treatment with the RNA synthesis inhibitor actinomycin D was shorter in HCC cells with PA2G4 knockdown than in corresponding control cells, indicating that PA2G4 increased the stability of FYN mRNA (Fig. 5A). Furthermore, RIP assay showed that PA2G4 bound with the mRNA of FYN (Fig. 5B).

To explore how PA2G4 stabilized the FYN mRNA, we overexpressed PA2G4 in HEK-293T cells by transducing pcDNA3.1-PA2G4 plasmids, and performed immunoprecipitation (IP) to enrich PA2G4-binding proteins in HEK-293T cells, followed by sliver staining (Fig. 5C). Liquid chromatography tandem-mass spectrometry (LC-MS) analysis was conducted with the PA2G4 immuno-precipitates to identify the binding partners of PA2G4. A total of 191 proteins were exclusively enriched in the PA2G4 immunoprecipitation (IP) groups and not in the IgG groups. Functional enrichment analysis (Additional file 1: Fig. S3B) and pathway enrichment analysis (Additional file 1: Fig. S3C) indicated that the binding protein of PA2G4 were mainly involved in clusters such as structural constituent of ribosome, RNA binding, nucleic acid binding and tight junction which was consistent with the roles of PA2G4 as the component of ribosomal complexes [9], RNA binding protein [7] and cell mobility mediator [7]. YTHDF2, an RNA-binding protein that affects the half-life of mRNAs, was found among the top 30 enriched binding partners of PA2G4 (Fig. 5D and Additional file 1: Table S5). The endogenous IP assay demonstrated that PA2G4 bound to YTHDF2 in both HEK-293T cells and HCC cells (Fig. 5E, F). An in-situ IF assay showed that PA2G4 colocalized with YTHDF2 in HCC cells (Fig. 5G). At the same time, neither overexpressing nor knocking down PA2G4 altered the expression of YTHDF2 in HCC cells (Additional file 1: Fig. S3D). Moreover, RIP assays in HCC cells showed that YTHDF2 bound with the mRNA of FYN as well (Fig. 5H).

### PA2G4 increased FYN expression in a YTHDF2-dependent manner

**Fig. 6:**
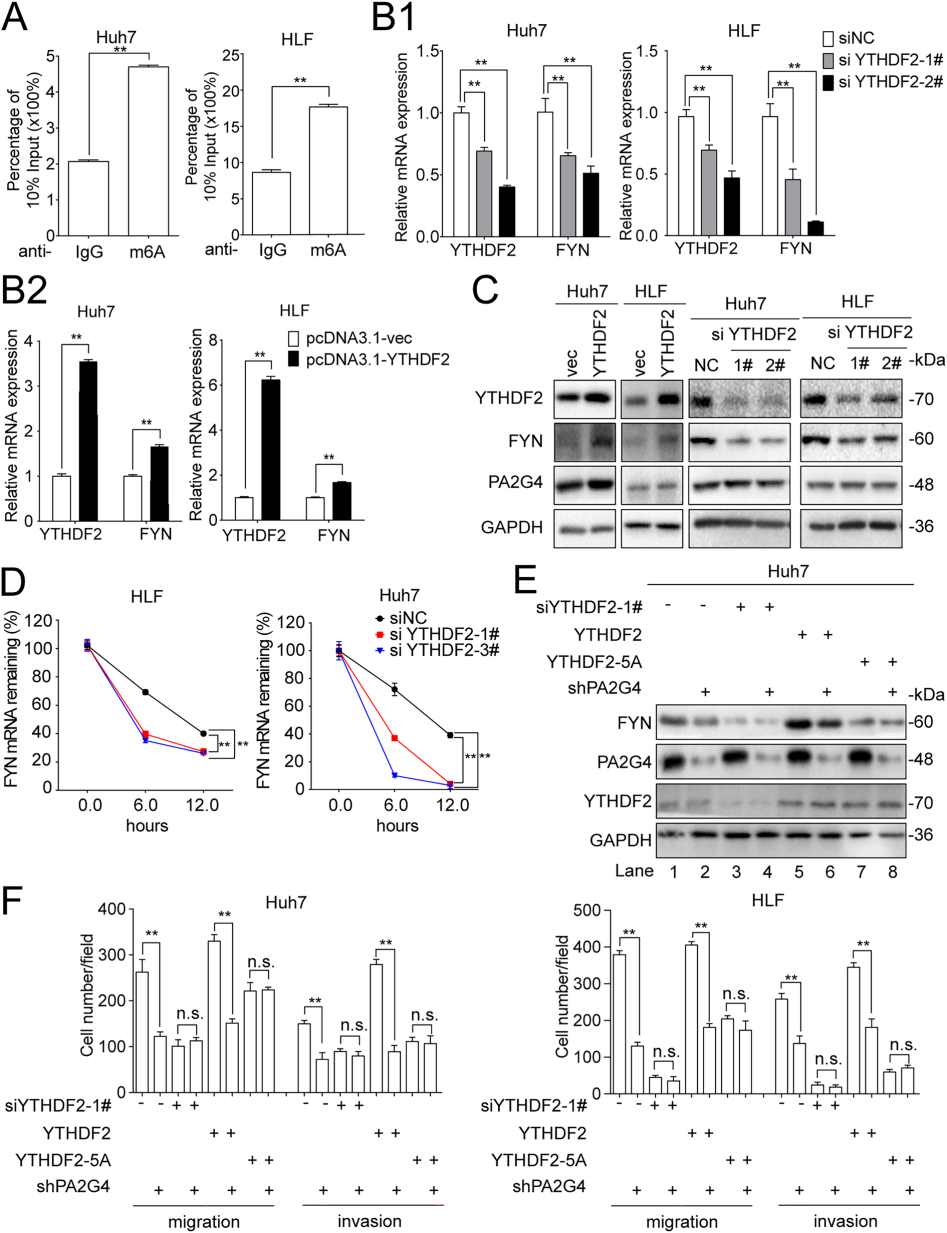
PA2G4 increases FYN expression in a YTHDF2-dependent manner. **A** MeRIP assay for detecting the m6A modification on the mRNA of FYN. Data was shown as percentage of FYN mRNA in anti-m6A precipitates against 10% input. **B** HCC cells were transfected with siRNA targeting YTHDF2 (**B1**) or pcDNA3.1-YTHDF2 (**B2**). qRT-PCR analyses were performed to evaluating the mRNA level of YTHDF2 and FYN 72 h after transfection. Data normalized to GAPDH and are shown as fold change to their respective control cells. **C** HCC cells were treated as in **B**, Western blot analyses of the indicated proteins. GAPDH as loading control. **D** HCC cells were transfected with siRNA targeting YTHDF2 for 72 h before administration of actinomycin D. qRT-PCR was performed to determine the relative FYN mRNA expression after treating with actinomycin D for the indicated time in each group. Data normalized to HPRT1 and are shown as percentage of the relative level in 0 h. **E** Huh7 cells with PA2G4 knocked down were transfected with siYTHDF2-1#, pcDNA3.1-YTHDF2 and pcDNA3.1-YTHDF2-5 A for 72 h. Western blot analysis of the indicated proteins. GAPDH as loading control. **F** Cells were treated as in **E**, tranwell assays were then performed to evaluate the cell migration and invasion abilities. Quantification of cells migrated and invaded. Data was shown as Mean ± SD. Two-tailed Student t test for (**A**, **B**, **D** and **F**). *p < 0.05, **p < 0.01. si: small interference; NC: negative control

Several potential m6A modification sites were predicted on the FYN mRNA with varying confidence (Additional file 1: Fig. S4A), and MeRIP assays demonstrated that FYN mRNA was m6A modified in HCC cells (Fig. 6A). Treatment with the m6A inhibitor DAA decreased FYN protein levels in a dose-dependent manner (Additional file 1: Fig. S4B), indicating the regulation of FYN expression by m6A modification.

At the same time, qRT-PCR and western blot analyses showed that YTHDF2 upregulated the mRNA and protein expression of FYN, without affecting the expression of PA2G4 protein (Fig. 6B, C). Knocking down YTHDF2 decreased the half-life of FYN mRNA (Fig. 6D). We then knocked down YTHDF2 in PA2G4 overexpressing Huh7 and HLF cells, and found that PA2G4 failed to upregulate FYN expression in the absence of YTHDF2 (lane 3 vs. lane 4) (Fig. 6E and Additional file 1: S4C). At the same time, recovering the expression of wild-type YTHDF2 expression (lane 5 vs. lane 6), but not of a catalytically inactive YTHDF2 mutant (YTHDF2-5 A) [42] (lane 7 vs. lane 8) rescued the regulation of FYN expression and cell mobility by PA2G4 (Fig. 6E, F and Additional file 1: Fig. S4C, 4D). These results indicated that PA2G4 promoted FYN expression and cell mobility via YTHDF2 in an m6A-catalytic manner.

### FYN mediated the pro-metastatic role of PA2G4

**Fig. 7:**
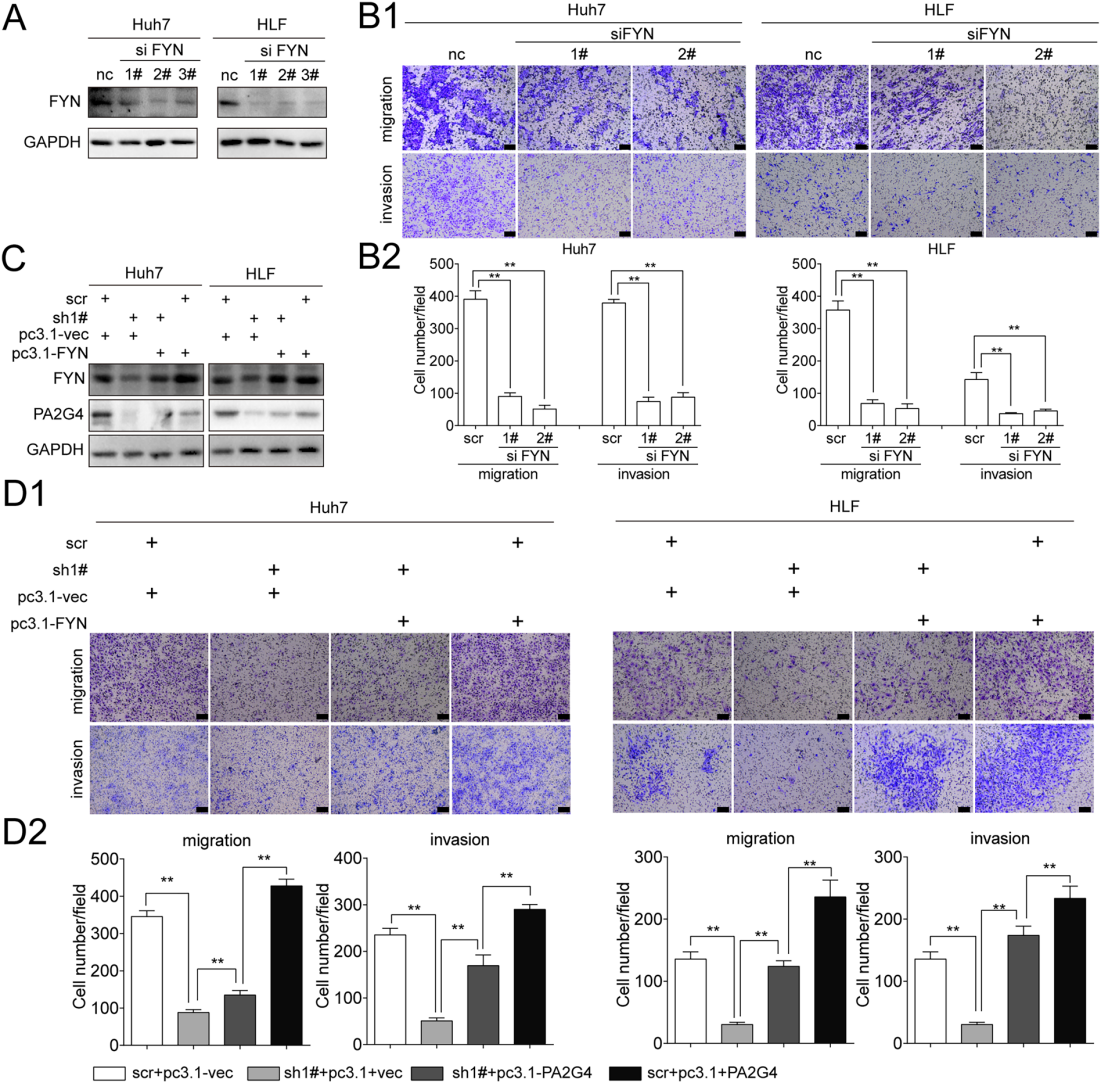
FYN mediates the pro-metastatic role of PA2G4. **A** Cells were transfected with siRNA targeting FYN for 72 h. Western blot analysis of the knocking down efficacy by siRNA. GAPDH as loading control. **B** Representative images (**B1**) and quantification (**B2**) of the migrated and invaded cells in the indicated groups. Scale bar, black bar, 100 μm. **C** Cells with PA2G4 stably knocked down were transfected with pcDNA3.1-FYN to transient overexpressing FYN. Western blot analysis of the indicated proteins. GAPDH as loading control. **D** Cells were treated as in **C**. Representative images (**D1**) and quantification (**D2**) of the migrated and invaded cells in the indicated groups. Scale bar, black bar, 100 μm. Data was shown as Mean ± SD. Two-tailed Student t test for (B2 and D2). *p < 0.05, **p < 0.01. pc3.1: pcDNA3.1

To determine whether FYN mediated the pro-metastatic effects of PA2G4, siRNA targeting FYN or pcDNA3.1-FYN was used to knock down or overexpress FYN in Huh7 and HLF cells (Fig. 7A and Additional file 1: Fig. S5A–5C). Transwell assays showed that FYN promoted the migration and invasion of HCC cells in vitro (Fig. 7B and Additional file 1: Fig. S5D). Moreover, rescue experiments were performed by recovering FYN expression in HCC cells with PA2G4 knockdown (Fig. 7C). The impaired migration and invasion of cells following PA2G4 knockdown cells could be rescued by overexpressing FYN (Fig. 7D).

### The expression of FYN was positively correlated with PA2G4 in HCC, and high expression of FYN predicted a poor prognosis

**Fig. 8:**
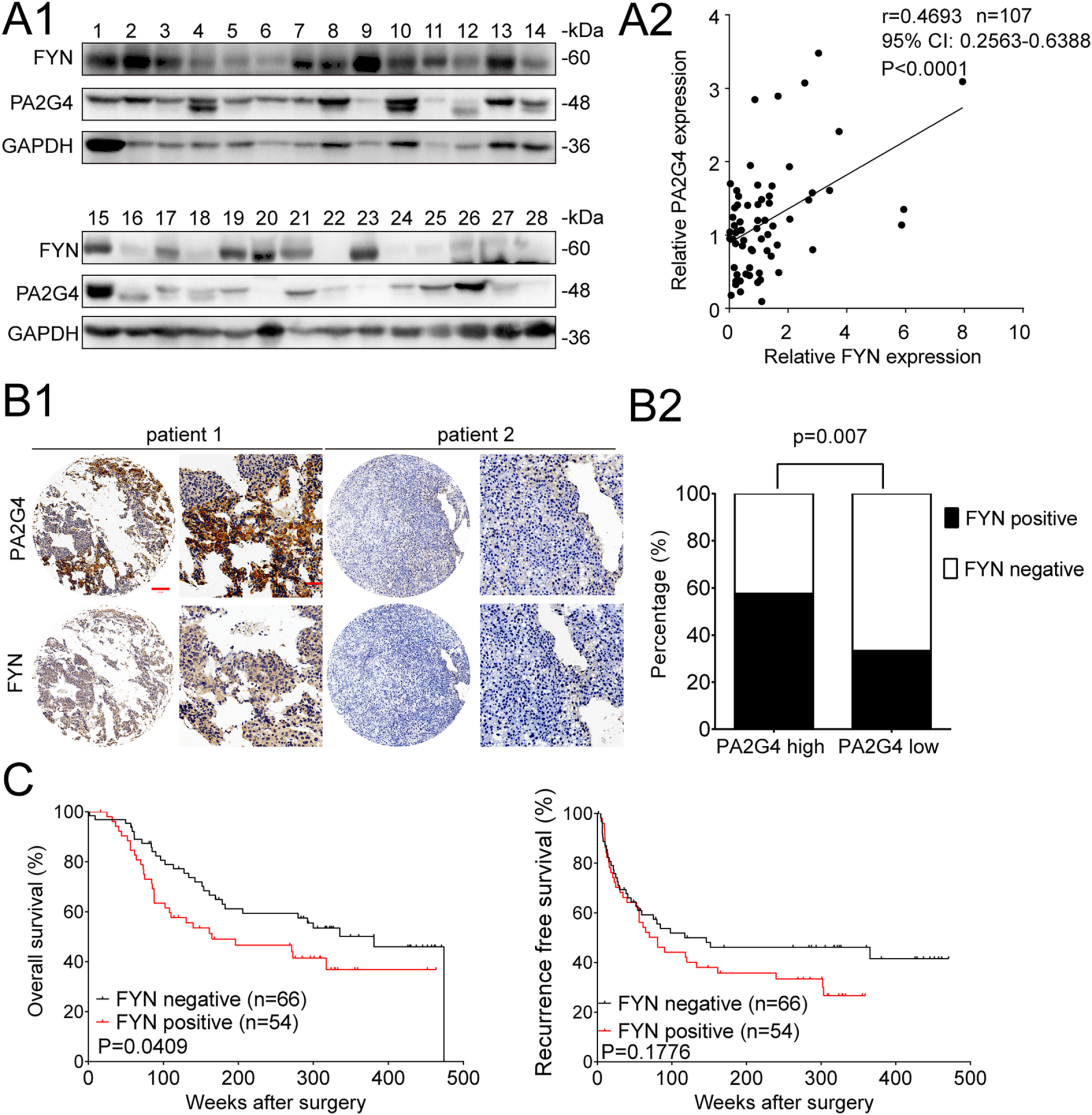
The expression of FYN is positively correlated with PA2G4 in HCC and high expression of FYN predicts poor prognosis. **A** The expression correlation between PA2G4 and FYN in HCC. Representative western blot bands of PA2G4 and FYN in 107 HCC tissues (**A1**). Relative PA2G4 and FYN expression level were determined by normalizing optical density to GAPDH. Correlation of relative PA2G4 and FYN expression in HCC specimens were presented by Pearson analysis (**A2**). **B** Representative IHC images of PA2G4 and FYN in HCC tissues (**B1**). Scale bar: red bar, 200 μm in the overview images; 50 μm in the magnified images. Chi-square analysis of the correlation between PA2G4 and FYN expression (**B2**). **C** Kaplan–Meier analysis for the overall survival and recurrence free survival of patients with different level of FYN. Pearson’s correlation test for (**A2**); Chi-square test for (**B2**); Log rank test for (**C**)

Western blot analysis of 107 HCC specimens demonstrated that the expression of FYN was positively associated with PA2G4, even across HCC samples in different BCLC stages (Fig. 8A and Additional file 1: Fig. S6 A, 6B). IHC staining for FYN and PA2G4 in HCC patient cohort 3 (Additional file 1: Table S6) further validated this correlation according to Chi-squared analysis (Fig. 8B and Additional file 1: Fig. S6C). At the same time, high expression of FYN was associated with microvascular invasion, a higher Child-Pugh score and advanced BCLC stage (Additional file 1: Table S7). Kaplan–Meier survival analysis showed that high expression of FYN predicted a shorter overall survival (Fig. 8C).

## Discussion

In the present study, we demonstrated that PA2G4 is a risk factor associated with poor prognosis in HCC patients. High PA2G4 expression confers HCC cells with increased metastatic ability by upregulating the expression of FYN, an EMT related pro-oncogene.

The role of PA2G4 has been intensively investigated in multiple cancer types. Nguyen et al. reported that PA2G4 promoted the proliferation of acute myelogenous leukemia (AML) cells by increasing the synthesis of rRNA and stability of proliferating cell nuclear antigen (PCNA) [15]. In colon cancer, PA2G4 was found to enhance rRNA synthesis by facilitating the function of TIF-90 and preventing its proteasomal degradation, further promoting cancer cell proliferation, colony formation, invasion and resistance to radiosensitivity [10]. However, it was also found to exert a tumor suppressor role in some cancers. In prostate cancer, PA2G4 was found to downregulate the expression of androgen receptor (AR) and AR-regulated genes, leading to lower incidence and weight of LNcaP tumors [43]. In thyroid cancer cells, PA2G4 inhibited proliferation, migration and invasion by increasing the expression of RAS protein activator like 1 (RASAL1). However, there were inconsistent reports on the role of PA2G4 in HCC. Hu et al. demonstrated that the expression of PA2G4 in HCC was significantly lower compared with ANTs, low expression of PA2G4 in HCC was associated with larger tumor size, advanced histological grade, higher ki67 score and shorter OS [25]. By contrast, Bao et al. showed that PA2G4 was highly expressed in HCC compared with normal tissues, and its expression was correlated with larger tumor size and shorter OS [24]. Interestingly, though HCC patients with higher PA2G4 levels displayed longer overall survival time, multivariate Cox analysis failed to conclude it as an independent risk factor for shorten OS, which may should be attributed for the relatively small body of patient number in the current research. PA2G4 exerts versatile functions in cancer cells [7], we doubted the possibility of its role as “housekeeping gene” in some cancer biological behaviors. Nevertheless, in our study, the mRNA and protein levels of PA2G4 were found significantly higher in HCC samples than in normal liver tissues evidenced by TCGA database and clinical specimens. Higher expression of PA2G4 was associated with shorter OS and RFS time, and was an independent risk factor for HCC recurrence. In addition, its relevance with HCC metastasis was proved by elevated expression level in HCC patients with extrahepatic metastases, as well as gain or loss function experiments in vivo and in vitro. These findings potentiated its applications as a diagnostic marker, prognostic index and therapeutic target in HCC.

The epithelial to mesenchymal transition of cancer cells modifies the expression of various genes that are crucial for metastatic properties [44, 45]. However, assessing the EMT status of cells is often confusing due to its complexity and the lack of a consensus definition. In 2020, a consensus was achieved for the guidelines and definition for studies on the EMT. The judgement of EMT status was recommended to be made on the basis of cellular properties, molecular markers and EMT-related transcriptional factors (EMT-TFs) [45]. At the same time, EMT process is actually a consistent dynamic process consisting of a wide spectrum of intermediary states, rather than a single binary decision [46]. The “partial EMT” or “hybrid EMT” was proposed and recognized as a useful concept for cells with mixed epithelial and mesenchymal phenotypes [46]. Additionally, changes in the expression of EMT markers are not necessarily consistent in cells with overexpression or knockdown of a specific gene [47]. In our study, we showed that PA2G4 enhanced the migration and invasion ability of cancer cells, induced a rearrangement of F-actin and modulated the expression of EMT-related molecular markers (E-cadherin, N-cadherin, occluding, Vimentin, α-SMA and ZO-1) as well as TFs (ZEB1, ZEB2 and Snail). At the same time, we noticed that these changes of molecular markers and TFs were dependent on the cellular context. Thus, we deduced that PA2G4 induced partial EMT in HCC cells.

Researches regarding the role and function of YTHDF2 in HCC are highly inconsistent and context dependent. Zhong et al. and Hou et al. reported that the expression of YTHDF2 was depressed in HCC compared with normal liver tissues [42, 48]. Hypoxia in HCC resulted in decreased expression of YTHDF2, and then induced aggressive transition of HCC cells by enhancing proliferation, migration, invasion abilities, together with neo-vascular formation and inflammation [42, 48]. At the same, numerous researches revealed YTHDF2 as an oncogenic promoter in HCC, evidenced by elevated expression in HCC compared with normal liver tissues and pro-metastatic effects in animal models [31, 49]. Specially, YTHDF2 was found to promote the liver cancer stem cell phenotype and lung metastasis by enhancing the translation of OCT4 mRNA [31], and was an independent risk factor for recurrence after hepatectomy [49]. In our research, PA2G4 was found to promote EMT of liver cancer cells by stabilizing mRNA of FYN in a YTHDF2 dependent manner, and was an independent risk factor for recurrence. These conclusions support YTHDF2 as an oncogene in HCC. From the perspective of molecular function, YTHDF2 was commonly taken as a mediator for mRNA decay [28], while emerging evidenced unveiled its multifaceted functions in RNA biology. YTHDF2 could preserve the m6A modification in the 5’UTR of mRNA and subsequentially promote its translation [30]. Also, YTHDF2 was found to stabilize MYC and VEGFA transcripts in glioblastoma stem cells in an m6A-dependent manner [32]. In the current study, we demonstrated YTHDF2 stabilized the mRNA of FYN. The m6A binding and recognizing amino acid residues of YTHDF2 were indispensable for this effect, implying this effect was m6A dependent. However, the determinates for decaying or stabilizing m6A modified transcripts by YTHDF2 still remained as a mystery, and may deserve further investigation. M6A modification was widely identified in eukaryotic cells, and the expression or activity of FYN was modulated by many signaling pathways [50]. The regulation of YTHDF2 on FYN may be not only dependent on stabilization of FYN mRNA, we observed that some upstream molecules of FYN were also regulated by YTHDF2 in HCC cells (data not shown). Nevertheless, our results demonstrated that YTHDF2 mediated the modulation of FYN by PA2G4 by stabilizing the mRNA of FYN.

## Conclusions

PA2G4 was highly expressed in HCC, and elevated expression of PA2G4 in HCC predicted poor prognosis. PA2G4 promoted the metastasis of HCC by inducing EMT through stabilizing FYN mRNA. YTHDF2 mediated the impact of PA2G4 on FYN in an m6A-catalytic dependent manner.

## Methods

### Patients and tissue specimens

Hepatocellular carcinoma (HCC) and adjacent non-tumor tissues were collected from HCC patients underwent hepatectomy at the Hepatic Surgery Center, Tongji Hospital of Huazhong University of Science and Technology (HUST) (Wuhan, China). 64 pairs of snap-frozen and 116 pairs of formalin fixed, paraffin-embedded (FFPE) tissues (cohort 1) were used for western blot and immunohistochemistry (IHC) analysis of PA2G4 expression, respectively. Another patient cohort (cohort 2) consisting of 57 HCC patients diagnosed as BCLC B, C or D stage with follow-up information of metastatic event was subjected to IHC analysis of PA2G4 expression. Additionally, 107 snap-frozen and 120 FFPE HCC tissues (cohort 3) were used to evaluated the expression correlation between PA2G4 and FYN in HCC. The clinical characteristics and survival/recurrence outcomes of HCC patients in cohort 1 and 3 with FFPE tissues were recorded and followed for further analysis. All procedures were approved by the Ethics Committee of Tongji Hospital, HUST and conducted according to the Declaration of Helsinki Principles. Prior written and informed consent were obtained from each patient.

### Cell lines and cell culture

HepG2, Huh7, HLF and HEK-293T were purchased from China Center for Type Culture Collection (CCTCC, Wuhan, China). MHCC97-H and HCC-LM3 were obtained from Liver Cancer Institute, Zhongshan Hospital, Fudan University, Shanghai, China. Hep3B and PLC/PRF/5 (ALEX) were purchased from cell bank of Chinese Academy of Sciences (Shanghai, China). The 4th generation of bone metastatic HCC cells (BM4-1, BM4-2 and BM4-3) were established in our previous study [41]. HCC cell lines were classified into early or advanced stage according to their morphology, invasive and metastatic potential [51]. Detailed characteristics of HCC cell lines used in this research were listed in Additional file 1: Table S4.

All cell lines were maintained in Dulbecco Modified Eagle Medium (DMEM) (Hyclone, UT, USA) supplemented with 10% fetal bovine serum (FBS) (Gibico) at 37 °C in a 5% CO_2_ cell incubator.

### Bioinformatic analysis and in silico prediction

Gene expression profiling interactive analysis (GEPIA) was applied to determine the mRNA expression pattern of PA2G4 in HCC [52]. Kaplan–Meier Plotter was used to evaluated the association of PA2G4 mRNA level with the overall survival (OS), recurrence free survival (RFS), progression free survival (PFS) and disease free survival (DSS) in HCC [53]. A sequence-based N6-methyladenosine (m6A) modification site predictor (SRAMP) was applied to predict the m6A modification on the mRNA of FYN using the full transcript model with RNA secondary structure analyzed [54].

### Animal experiments

BALB/c-nude mice (male, 4 weeks old) were purchased from Beijing HFK Bioscience Co. Ltd. Mice were maintained at specific pathogen free (SPF) conditions, and accommodated for one week after arrival before applying for animal experiments. All animal experiments were approved by the Ethics Committee of Tongji Hospital, HUST (TJH-202103010). The whole procedure was in accordance with the “Guide for the Care and Use of Laboratory Animals” (NIH publication 86–23 revised 1985). For lung metastasis model, 1 × 10^6^ HCC cells suspended in 100 µl serum free DMEM were injected via the tail vein of nude mice. After 2 months of injection, mice were sacrificed and lungs were resected for H&E staining to calculate metastatic foci.

### Dual luciferase reporter assay

PGL4.17-vector or PGL4.17-FYN-promoter was co-transfected with PRL plasmids into PA2G4 knocked down HCC cells. Renilla and firefly luciferase activities were determined using Dual Luciferase® Reporter 1000 Assay System (Promega, Madison, WI, USA) by GloMax 20/20 Luminometer according to the manufacturer’s instructions. Firefly luciferase values were normalized against Renilla luciferase activity, and the ratio of firefly/renilla luciferase activity was presented.

### RNA binding protein immunoprecipitation assay (RIP)

RIP assays were performed as previously described with some modifications [28]. Briefly, cells in two 10 cm dishes were harvested in 500 µl pre-cold RIP lysis buffer (50 mM Tris-HCl pH 7.4, 150 mM NaCl, 1 mM EDTA, 1 mM dithiothreitol, 0.5% NP-40, 0.1U/µl RiboLock RNase Inhibitor (Thermo Fisher Scientific) and EDTA-free protease inhibitor cocktail (Roche)). Lysate was placed on ice for 10 min and then centrifuged at 14,000*g* for 15 min at 4 °C. Five hundred microliters of supernatant was subjected to 30 µl protein G conjugated agarose beads (GE Healthcare Life Sciences) complemented with either 5 µg anti-PA2G4 (15348-1-AP) or anti-YTHDF2 antibody (24744-1-AP, Proteintech) or non-specific rabbit IgG (2729, CST) and rotated for 3 h at 4 °C. Next, the beads were washed with 1 ml pre-cold lysis buffer for eight times, and RNA was isolated from the beads using Trizol. qRT-PCR was used to measure the RNA abundance of FYN mRNA in each group. The 2^−ΔCT^ was calculated and was normalized to the 2^−ΔCT^ of 10% input.

### M6A immunoprecipitation (MeRIP)

RIP assays were performed as previously described with some modifications[28]. Total RNA from two 10 cm dishes was used for each IP reaction in 500 µl IP buffer (10 mM Tris-HCl pH 7.4, 150 mM NaCl, 0.1% NP-40, 0.4 U/µl RiboLock RNase Inhibitor and 2 mM Ribonucleoside vanadyl complexes) with 5 µg of either m6A-specific antibody (ab151230, Abcam) or non-specific rabbit IgG (2729, CST). Fifty microliters of pre-washed protein G conjugated agarose beads (GE Healthcare Life Sciences) were added into the IP samples and rotated gently for 4 h at 4 °C. The beads were washed with 1 ml IP buffer for six to nine times and the bound RNA was purified from the beads using Trizol. qRT-PCR was applied to determine the enrichment of m6A modified FYN mRNA in each group. The 2^−ΔCT^ was calculated and was normalized to the 2^−ΔCT^ of 10% input.

### Statistical analysis

Statistical analyses were performed using Prism 7.0 (GraphPad Software, La Jolla, CA, USA) or SPSS 13.0 (SPSS, Chicago, IL, USA) software. The results were presented as the mean ± standard deviation (M ± SD). Quantitative data were analyzed by Two tailed Student t test or Pearson’s correlation test. Categorical data were analyzed by Chi-square test or Fisher’s exact test. Kaplan-Meier analysis was used to assess the survival between subgroups, median IHC score was used to divided patients into high/low expression group. Cox proportional hazards model (Forward stepwise, likely ratio) was used to determine the risk factors of survival and recurrence. Variables with P value < 0.05 in univariate Cox analysis were further included for multivariate Cox analysis to test their dependence. P value < 0.05 was considered statistically significant.

## Supplementary Information

**Additional file 1.** Figure S1–S6. Tables 1–7. Materials and methods.

## Data Availability

The datasets used and/or analyzed during the current study are available from the corresponding author on reasonable request.

## Abbreviations

aa: Amino acids
AML: Acute myelogenous leukemia
ANTs: Adjacent normal tissue
AR: Androgen receptor
BCLC: Barcelona Clinic Liver Cancer
CI: Confidential index
DFS: Disease free survival time
EBP1: ErbB3-binding protein 1
EMT: Epithelial to mesenchymal transition
FFPE: Paraffin-embedded
FYN: FYN proto-oncogene
SFK: Src Family tyrosine kinase
F-actin: Actin fibers
GO: Gene ontology
HR: Hazard ratio
HCC: Hepatocellular carcinoma
HER2: Human epidermal growth factor receptor 2
IF: Immunofluorescence
IHC: Immunohistochemistry
IP: Immunoprecipitation
KEGG: Kyoto Encyclopedia of Genes and Genomes
MeRIP: Anti-m6a immunoprecipitation
m6A: N6-methyladenosine
OS: Shorter overall survival time
PA2G4: Proliferation associated protein 2G4
PCNA: Proliferating cell nuclear antigen
PFS: Progression free survival time
qRT-PCR: Quantitative real time-PCR
RASAL1: RAS protein activator like 1
RFS: Recurrence free survival time
RIP: RNA binding protein immunoprecipitation assay
RNA-Seq: RNA sequencing
TCGA: The Cancer Genome Atlas
UTR: Translational region
YTHDF2: YTH N6-methyladenosine RNA binding protein 2
