# Supplementary material for: PA2G4 promotes the metastasis of hepatocellular carcinoma by stabilizing FYN mRNA in a YTHDF2-dependent manner
Source: Cell Biosci. 2022 May 7;12:55. doi: 10.1186/s13578-022-00788-5 (PMC9080163; doi:10.1186/s13578-022-00788-5)
Supplement: Supplementary file 1 — Additional file 1. Figure S1–S6. Tables 1–7. Materials and methods. [file 13578_2022_788_MOESM1_ESM.docx]

**Supplementary figures and figure legends:**

**Supplementary figure 1.**

**
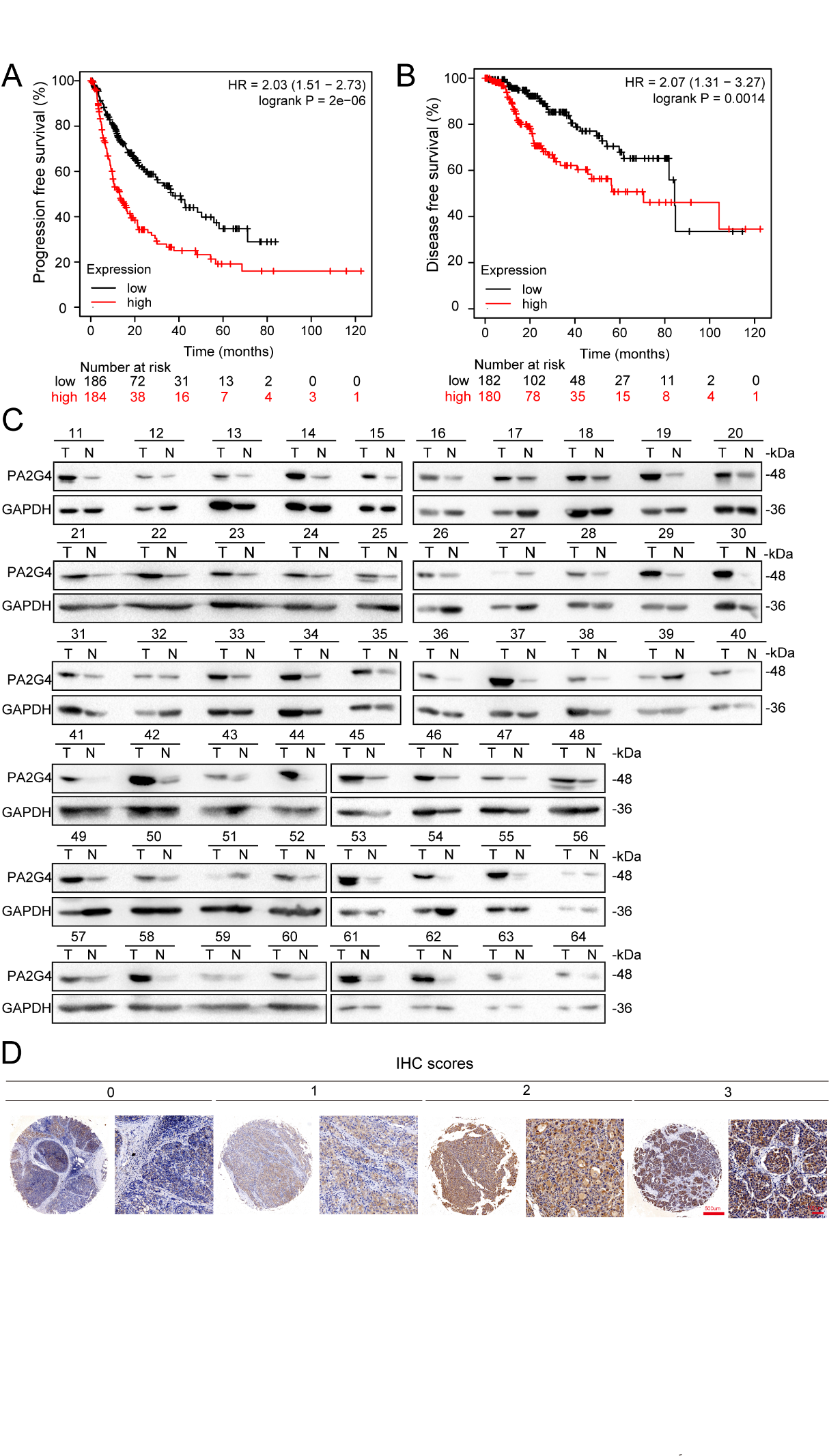
**

**Supplementary figure 1.** PA2G4 is upregulated in HCC and high expression of PA2G4 predicts poor prognosis. (A, B) Kaplan-Meier analysis of progression survival (A) and disease free survival (B) of HCC patients with different expression of PA2G4 in TCGA database analyzed by Kaplan-Meier Plotter. (C) Western blot analysis of PA2G4 expression in paired HCC specimens. (D) Representative IHC images of PA2G4 with the indicated staining intensity. Scale bar: red bar, 500 μm in the overview images; 100 μm in the magnified images. Log rank test for (A and B). Abbreviation: T, tumor; N, adjacent non-tumorous tissue.

**Supplementary figure 2.**


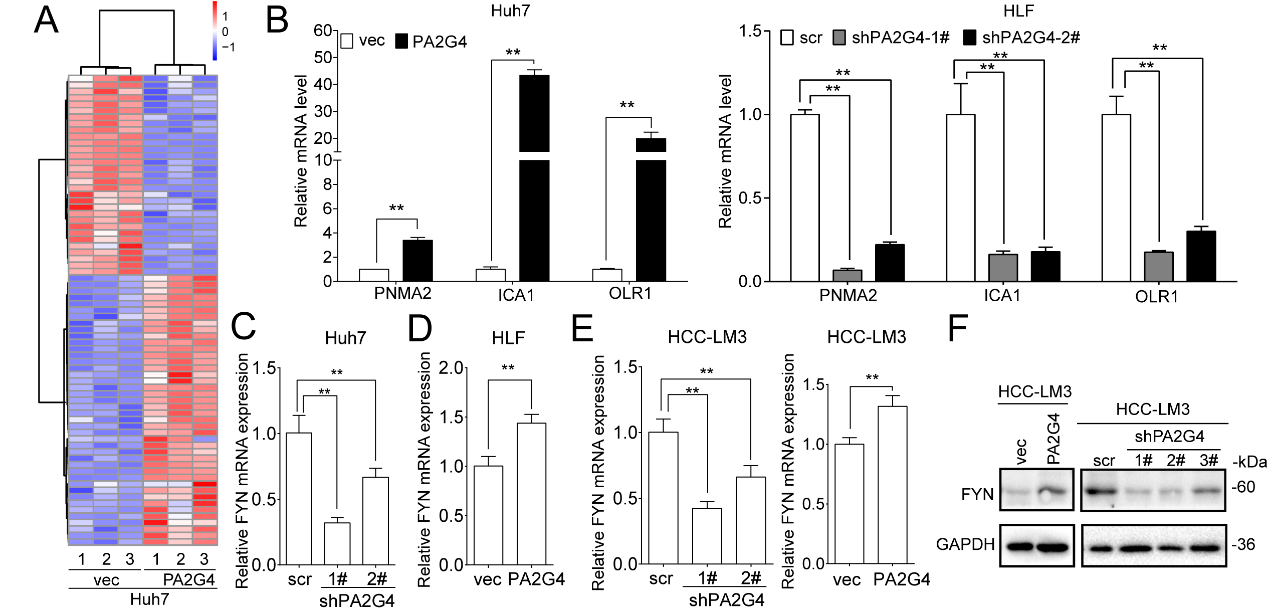


**Supplementary figure 2.** FYN is a downstream effector of PA2G4 in HCC. (A) Heatmap of the differentially expressed genes between PA2G4 overexpressed Huh7 cells and its control cells. Triplicates in each group were applied for RNA-Seq. (B) qRT-PCR analysis of the indicated genes in Huh7 or HLF cells with PA2G4 overexpressed or knocked down. (C, D) qRT-PCR analysis of FYN expression in Huh7 cells with PA2G4 knocked down (C) and in HLF cells with PA2G4 overexpression (D). (E, F) qRT-PCR (E) and western blot (F) analysis of FYN expression in HCC-LM3 cells with PA2G4 overexpression or knocked down. GAPAH as loading control. Data normalized to GAPDH and are shown as fold change to their respective control cells in (B, C, D and E). Data was shown as Mean±SD. Two-tailed Student t test for (B-E). *, p<0.05, **, p<0.01. Abbreviation: vec, vector; scr, scramble; sh, short hairpin.

**Supplementary figure 3.**


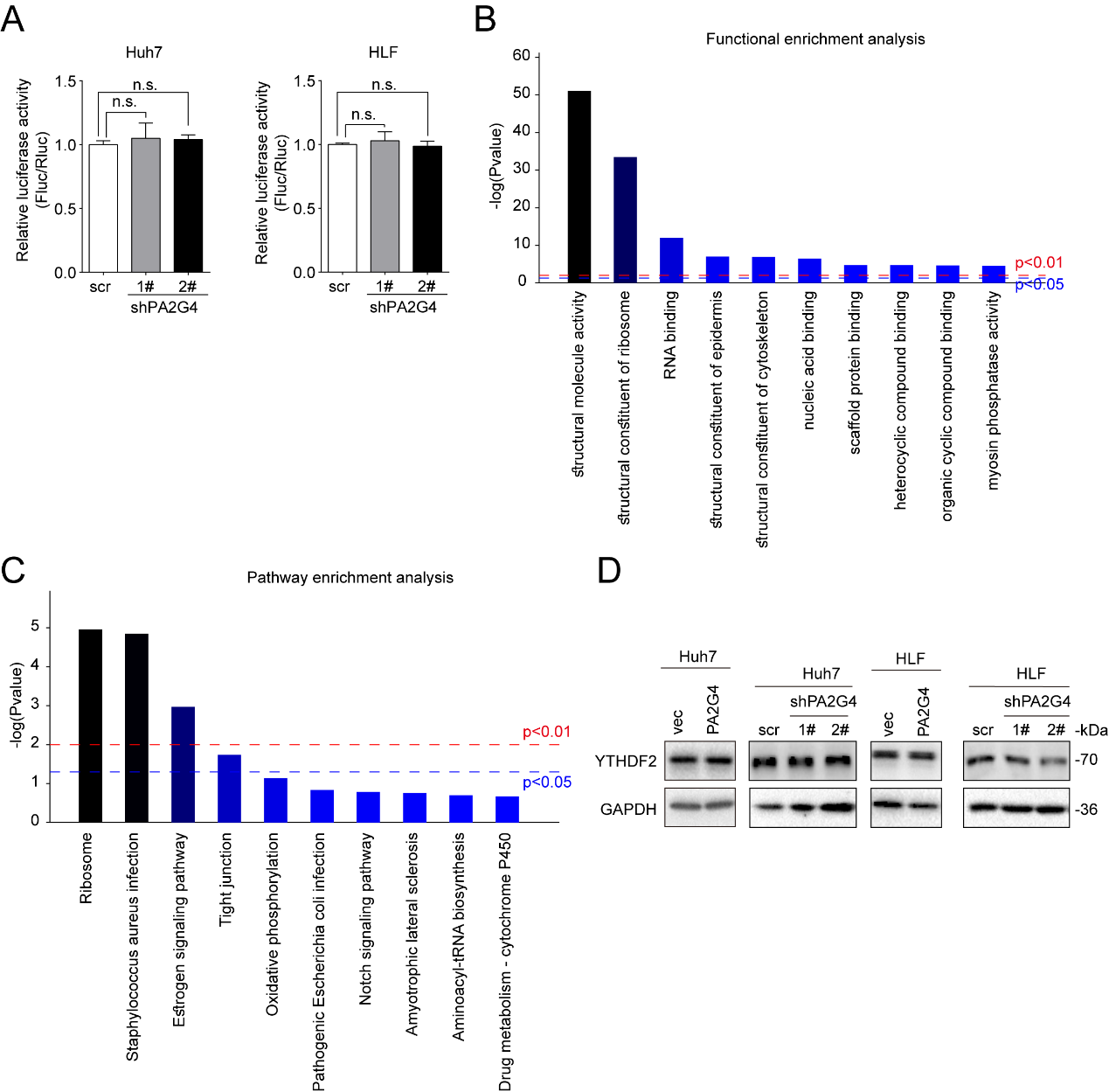


**Supplementary figure 3.** YTHDF2 is an endogenous binding patterner of PA2G4. (A) Dual luciferase activity assay for the FYN transcription activities in PA2G4 knocked down cells. (B, C) Top 10 terms of functional enrichment analysis (B) and KEGG analysis (C) of the inclusively proteins enriched by anti-PA2G4 antibody. (D) Western blot analysis of YTHDF2 expression in HCC cells with different level of PA2G4. GAPDH as loading control. Data was shown as Mean±SD. Two-tailed Student t test for (A, B and C). n.s., no significance. Abbreviation: Fluc, firefly luciferase; Rluc, renilla luciferase.

**Supplementary figure 4.**


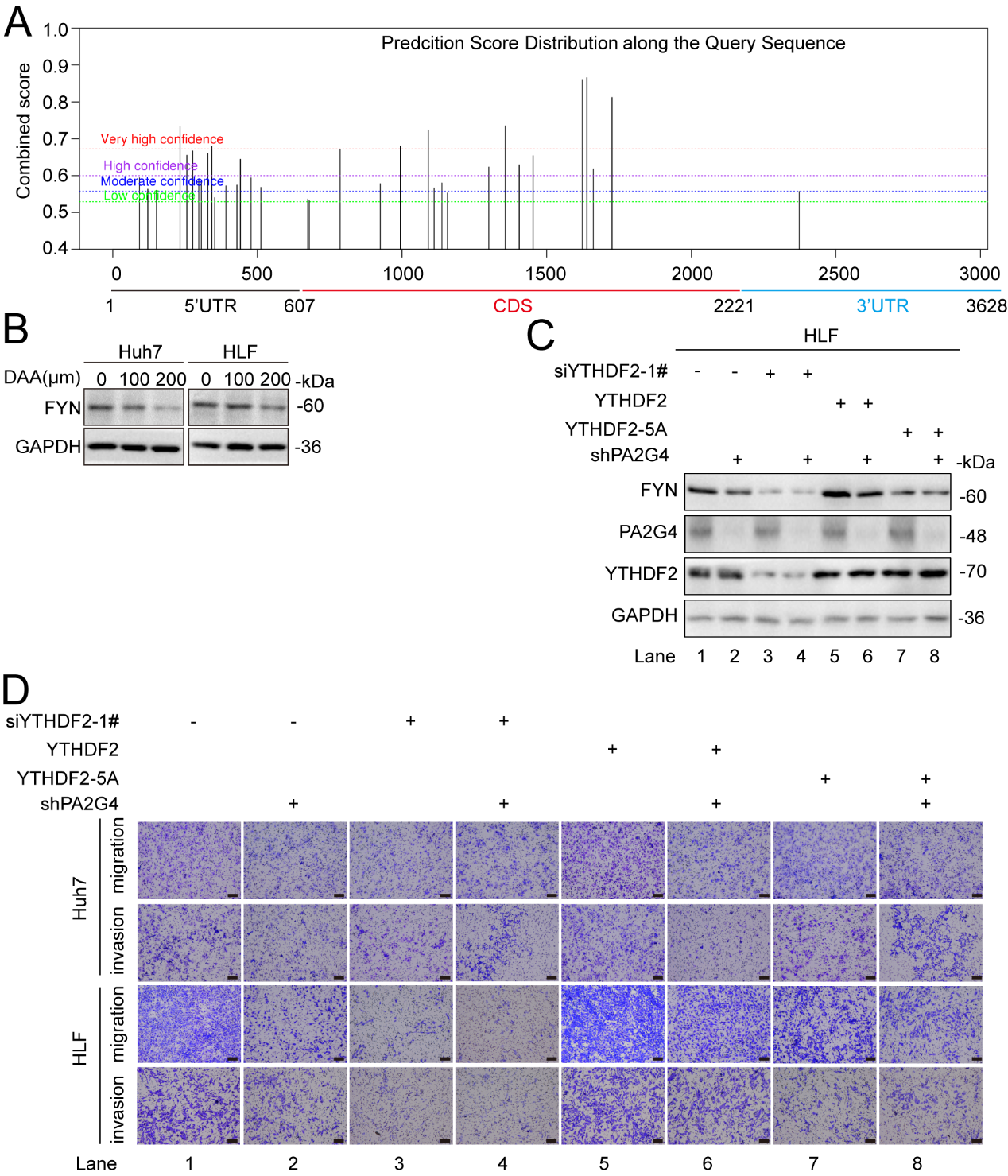


**Supplementary figure 4.** PA2G4 increases FYN expression in a YTHDF2-dependent manner. (A) The m6A modification in FYN mRNA predicted by the SRAMP website. (B) Cells were treated with DAA at the indicate concentration for 24 hrs. Western blot analysis of FYN expression. (C) HLF cells with PA2G4 knocked down were transfected with siYTHDF2-1#, pcDNA3.1-YTHDF2 and pcDNA3.1-YTHDF2-5A for 72 hrs. Western blot analysis of the indicated proteins. (D) Cells were treated as in (C), tranwell assays were then performed to evaluate the cell migration and invasion abilities. Representative images of cell migration and invasion. GAPDH as loading control in (B and C). Data was shown as Mean±SD.

**Supplementary figure 5.**

**
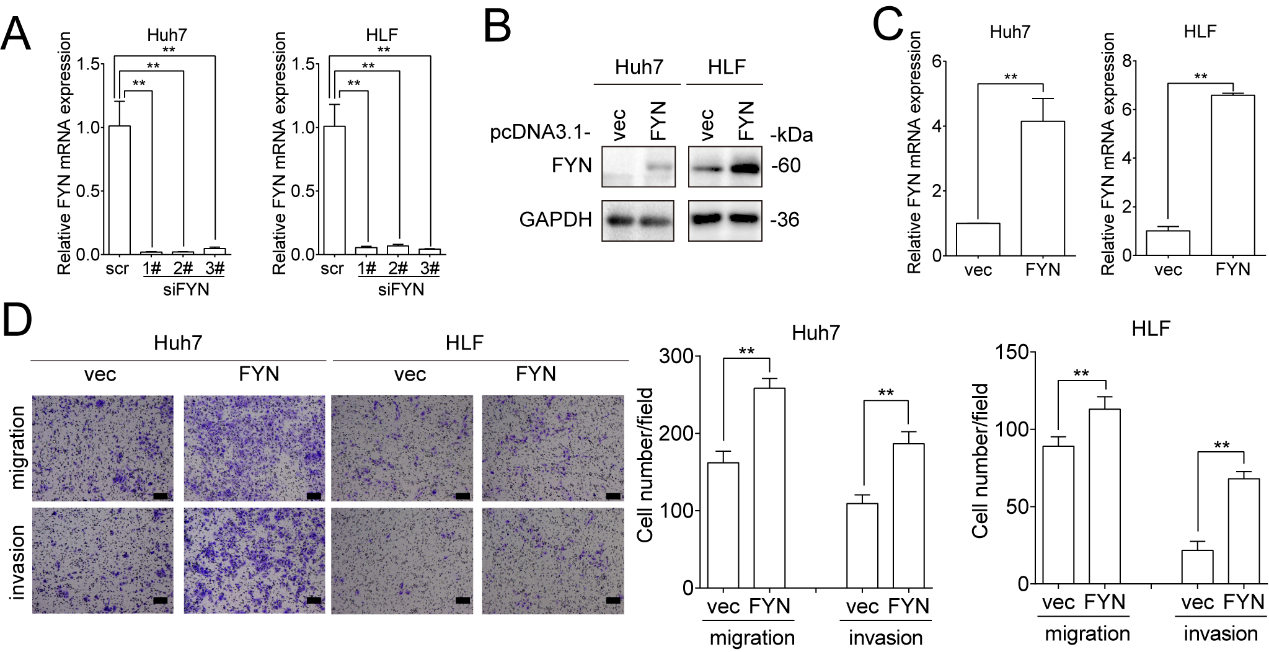
**

**Supplementary figure 5.** FYN mediates the pro-metastatic role of PA2G4. (A) qRT-PCR analysis of the knocking down efficacy by siRNA targeting FYN in Huh7 and HLF cells. Date normalized to GAPDH and are shown as fold change to their respective negative control cells. (B, C) Huh7 and HLF cells were transfected with pcDNA3.1-FYN or empty control pcDNA3.1-vector plasmids. Western blot (B) and qRT-PCR (C) analysis were then performed to evaluate FYN overexpression efficacy. (D) Representative images and quantification of cells migrated and invaded in the indicated groups. Data was shown as Mean±SD. Two-tailed Student t test for (A, C and D). *, p<0.05, **, p<0.01.

**supplementary figure 6.**


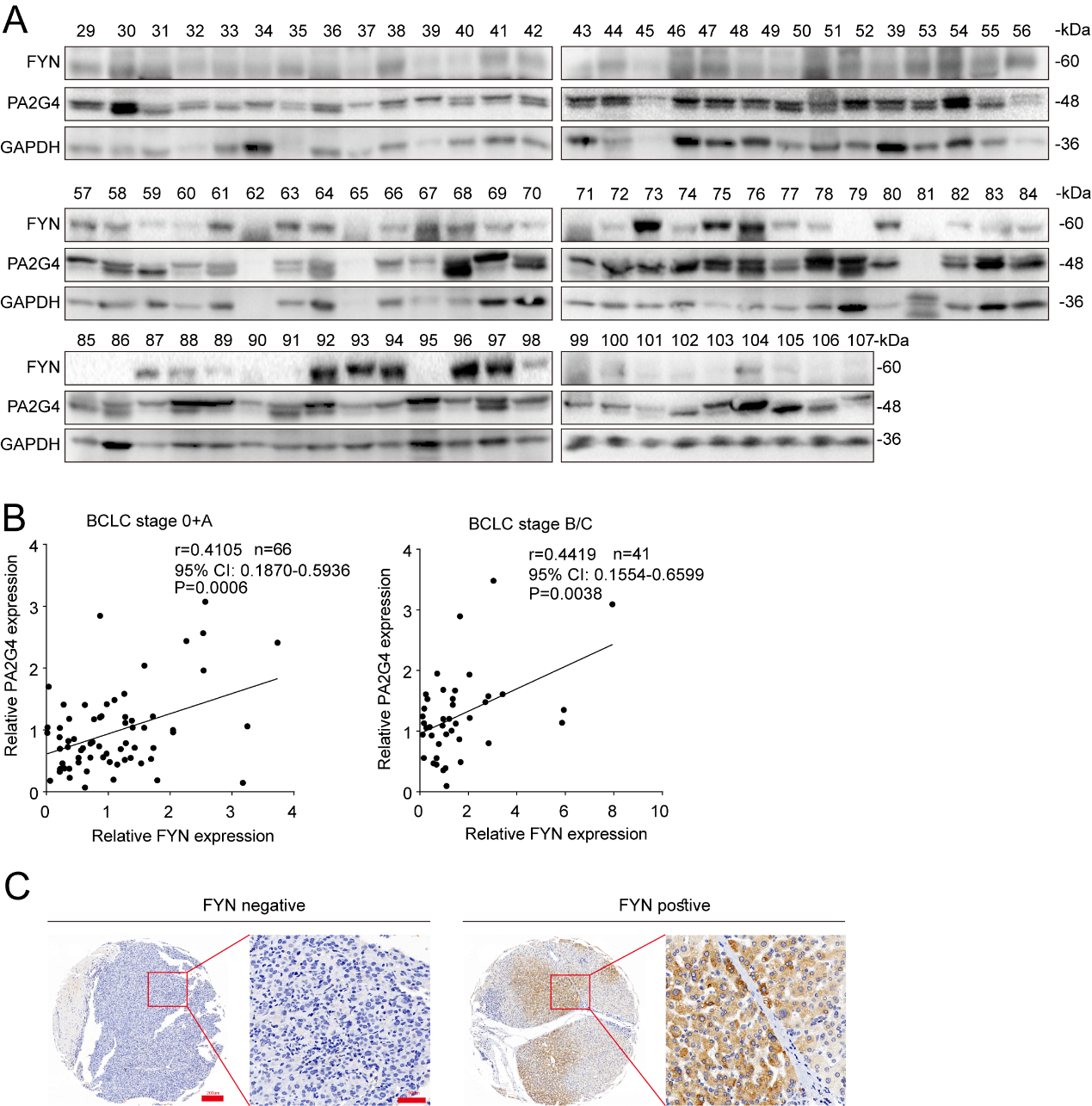


**Supplementary figure 6.** The expression of FYN is positively correlated with PA2G4 in HCC and high expression of FYN predicts poor prognosis. (A) Western blot analysis of FYN and PA2G4 in 107 HCC tissues (the additional 28 HCC tissues were presented in figure 8A1). (B) The correlations between PA2G4 and FYN expression level in HCC patient cohort 3 across different BCLC stages were determined Pearson analysis. (C) Representative IHC images of FYN positive and FYN negative. Scale bar, red bar, 200 μm in the overview images; 50 μm in the magnified images. Pearson’s correlation test for (B).

**Supplementary tables**

**Supplementary table 1. Clinicopathologic characteristics of patients with hepatocellular carcinoma in cohort1.**

| **Clinicopathological**  **variables** | **Number**  **n=116** | **Percentage (%)** |  |
| --- | --- | --- | --- |
|  |  |  |  |
| **Gender** |  |  |  |
| Male | 94 | 81.03 |  |
| Female | 22 | 18.97 |  |
| **Age** |  |  |  |
| ≤50 | 54 | 46.55 |  |
| >50 | 62 | 53.45 |  |
| **AFP (μg/L)** |  |  |  |
| ≤20 | 39 | 33.62 |  |
| >20 | 77 | 66.38 |  |
| **ALT(U/L)** |  |  |  |
| ≤41 | 83 | 71.55 |  |
| >41 | 33 | 28.45 |  |
| **AST(U/L)** |  |  |  |
| ≤40 | 91 | 78.45 |  |
| >40 | 25 | 21.55 |  |
| **GGT(U/L)** |  |  |  |
| ≤71 | 74 | 63.79 |  |
| >71 | 42 | 36.21 |  |
| **ALP(U/L)** |  |  |  |
| ≤130 | 98 | 85.48 |  |
| >130 | 18 | 15.52 |  |
| **HBV** |  |  |  |
| Negative | 15 | 12.93 |  |
| Positive | 101 | 87.06 |  |
| **HCV** |  |  |  |
| Negative | 114 | 98.28 |  |
| Positive | 2 | 1.72 |  |
| **Cirrhosis** |  |  |  |
| No | 12 | 10.34 |  |
| Yes | 104 | 89.66 |  |
| **Ascites** |  |  |  |
| No | 94 | 80.03 |  |
| Yes | 22 | 19.97 |  |
| **Tumor size** |  |  |  |
| ≤5 | 61 | 52.59 |  |
| >5 | 55 | 47.41 |  |
| **Tumor number** |  |  |  |
| Single | 95 | 81.9 |  |
| Multiple | 21 | 18.1 |  |
| **Tumor encapsulation** |  |  |  |
| None | 47 | 40.52 |  |
| Complete | 69 | 59.48 |  |
| **Macrovascular invasion** | |  |  |
| No | 99 | 85.34 |  |
| Yes | 17 | 14.66 |  |
| **Microvascular invasion** |  |  |  |
| No | 76 | 65.52 |  |
| Yes | 40 | 34.48 |  |
| **Satellite nodules** |  |  |  |
| No | 84 | 72.41 |  |
| Yes | 32 | 27.59 |  |
| **Extrahepatic metastasis** | |  |  |
| Negative | 112 | 96.55 |  |
| Positive | 4 | 3.45 |  |
| **Child-Pugh** |  |  |  |
| A | 106 | 91.38 |  |
| B | 10 | 8.62 |  |
| **BCLC stage** |  |  |  |
| 0+A | 82 | 70.69 |  |
| B+C | 34 | 29.31 |  |

**Supplementary table 2. Correlation between relative PA2G4 expression and clinicopathologic characteristics of HCC patients in cohort1 (n= 116).**

| **Clinicopathological**  **variables** | **Relative PA2G4 expression** | | **P value** |
| --- | --- | --- | --- |
|  | **Low (n=54)** | **High (n=62)** |  |
| **Gender** |  |  |  |
| Male | 42 | 52 | 0.404 |
| Female | 12 | 10 |  |
| **Age** |  |  |  |
| ≤50 | 24 | 29 | 0.802 |
| >50 | 30 | 33 |  |
| **AFP (μg/L)** |  |  |  |
| ≤20 | 18 | 21 | 0.951 |
| >20 | 36 | 41 |  |
| **ALT (U/L)** |  |  |  |
| ≤41 | 40 | 43 | 0.574 |
| >41 | 14 | 19 |  |
| **AST (U/L)** |  |  |  |
| ≤40 | 43 | 48 | 0.773 |
| >40 | 11 | 14 |  |
| **GGT (U/L)** |  |  |  |
| ≤71 | 36 | 38 | 0.548 |
| >71 | 18 | 24 |  |
| **ALP (U/L)** |  |  |  |
| ≤130 | 44 | 54 | 0.405 |
| >130 | 10 | 8 |  |
| **HBV** |  |  |  |
| Negative | 10 | 6 | 0.168 |
| Positive | 44 | 56 |  |
| **HCV** |  |  |  |
| Negative | 52 | 62 | 0.215* |
| Positive | 2 | 0 |  |
| **Cirrhosis** |  |  |  |
| No | 8 | 4 | 0.14* |
| Yes | 46 | 58 |  |
| **Ascites** |  |  |  |
| No | 47 | 47 | 0.124 |
| Yes | 7 | 15 |  |
| **Tumor size** |  |  |  |
| ≤5 | 27 | 36 | 0.384 |
| >5 | 27 | 26 |  |
| **Tumor number** |  |  |  |
| Single | 48 | 47 | 0.068 |
| Multiple | 6 | 15 |  |
| **Tumor encapsulation** |  |  |  |
| None | 17 | 30 | 0.064 |
| Complete | 37 | 32 |  |
| **Macrovascular invasion** |  |  |  |
| No | 46 | 53 | 0.964 |
| Yes | 8 | 9 |  |
| **Microvascular invasion** |  |  |  |
| No | 40 | 36 | 0.07 |
| Yes | 14 | 26 |  |
| **Satellite nodules** |  |  |  |
| No | 42 | 42 | 0.228 |
| Yes | 12 | 20 |  |
| **Extrahepatic metastasis** |  |  |  |
| Negative | 54 | 58 | 0.165* |
| Positive | 0 | 4 |  |
| **Child-Pugh** |  |  |  |
| A | 51 | 55 | 0.272* |
| B | 3 | 7 |  |
| **BCLC stage** |  |  |  |
| 0+A | 43 | 39 | **0.048** |
| B+C | 11 | 23 |  |

Chi-square test was used to analyze categorical data; *, Fisher exact test.

**Supplementary table 3. Univariate and multivariate analysis of factors associated with survival and recurrence of 116 HCC patients in cohort 1.**

|  | **Survival** | | | | | | |
| --- | --- | --- | --- | --- | --- | --- | --- |
|  | **Univariate analysis** | | | **Multivariate analysis** | | | |
|  | **HR** | **95%CI** | **P value** | | **HR** | **95%CI** | **P value** |
| Gender (male vs female) |  |  | 0.248 | |  |  |  |
| Age (>50 vs ≤50) |  |  | 0.403 | |  |  |  |
| Serum AFP (>20 vs ≤20ug/L)  ALT (>75 vs ≤75 U/L)  GGT (>54 vs ≤54 U/L)  HBV (positive vs negative)  HCV (positive vs negative)  Cirrhosis (yes vs no) | 2.262  2.117 | 1.157-4.422  1.194-3.755 | **0.017**  0.293  **0.010**  0.105  0.286  0.734 | |  |  |  |
| Tumor size (>5 vs ≤5 cm)  Tumor number (multiple vs single) | 2.748 | 1.494-5.053 | 0.312  **0.001** | |  |  |  |
| Tumor encapsulation (none vs complete) | 3.185 | 1.792-5.650 | **0.000** | | 2.070 | 1.111-3.846 | **0.022** |
| Macrovascular invasion (yes vs no)  Microvascular invasion (yes vs no)  Satellite nodules (yes vs no)  Extrahepatic metastasis (yes vs no)  Child-Pugh (B vs A) | 3.971  2.091  3.516  7.353 | 2.086-7.557  1.196-3.657  2.002-6.176  2.2491-21.702 | **0.000**  **0.010**  **0.000**  **0.000**  0.745 | | 2.460  2.522  4.905 | 1.221-4.954  1.378-4.617  1.596-15.078 | **0.012**  **0.003**  **0.006** |
| BCLC stage (B+C vs 0+A)  PA2G4 expression (high vs low) | 3.041  1.849 | 1.735-5.331  1.049-3.258 | **0.000**  **0.034** | |  |  |  |

|  | **Recurrence** | | | | | | |
| --- | --- | --- | --- | --- | --- | --- | --- |
|  | **Univariate analysis** | | | **Multivariate analysis** | | | |
|  | **HR** | **95%CI** | **P value** | | **HR** | **95%CI** | **P value** |
| Gender (male vs female) | 2.469 | 1.182-5.155 | 0.016 | | 2.494 | 1.182-5.263 | **0.016** |
| Age (>50 vs ≤50) |  |  | 0.195 | |  |  |  |
| Serum AFP (>20 vs ≤20ug/L)  ALT (>75 vs ≤75 U/L)  GGT (>54 vs ≤54 U/L)  HBV (positive vs negative)  HCV (positive vs negative)  Cirrhosis (yes vs no) | 1.659  1.815 | 0.998-2.756  1.136-2.902 | **0.051**  0.240  **0.013**  0.311  0.156  0.351 | |  |  |  |
| Tumor size (>5 vs ≤5 cm)  Tumor number (multiple vs single) | 2.433  3.275 | 1.530-3.868  1.92-5.588 | **0.000**  **0.000** | | 2.553 | 1.555-4.193 | **0.000** |
| Tumor encapsulation (none vs complete) | 2.336 | 1.468-3.717 | **0.000** | |  |  |  |
| Macrovascular invasion (yes vs no)  Microvascular invasion (yes vs no)  Satellite nodules (yes vs no)  Extrahepatic metastasis (yes vs no)  Child-Pugh (B vs A) | 2.974  2.544  2.564  4.366 | 1.664-5.314  1.596-4.055  1.579-4.163  1.559-12.227 | **0.000**  **0.000**  **0.000**  **0.005**  0.422 | | 1.936  2.165 | 1.189-3.155  1.245-3.763 | **0.008**  **0.006** |
| BCLC stage (B+C vs 0+A)  PA2G4 expression (high vs low) | 3.445  1.736 | 2.131-5.569  1.093-2.757 | **0.000**  **0.020** | | 1.651  1.760 | 0.940-2.899  1.085-2.856 | **0.081**  **0.022** |

Bold, p<0.05

**Supplementary table 4. Characteristics of HCC cells lines.**

| **Cell line** | **Morphology** | **Differentiated grade** | **Biological Characteristics** |
| --- | --- | --- | --- |
| HepG2(1) | Epithelial | Well-differentiated | Non-invasive |
| Hep3B(1) | Epithelial | Well-differentiated | Non-invasive |
| Huh7(1) | Epithelial | Well-differentiated | Low-metastatic |
| PLC/PRF/5(2) | Epithelial | N.A. | Non-invasive |
| HLF(2) | Epithelial | N.A. | Invasive |
| MHCC97-H(1) | Epithelial | Edmonson grade II | Metastatic |
| HCC-LM3(1) | Epithelial | Edmonson grade II | Metastatic, relatively low bone metastatic |
| BM4-1(3) | Epithelial | N.A. | Metastatic, relatively moderate bone metastatic |
| BM4-2(3) | Epithelial | N.A. | Metastatic, relatively high bone metastatic |
| BM4-3(3) | Epithelial | N.A. | Metastatic, relatively high bone metastatic |

Abbreviations: N.A., not available/not reported.

**Supplementary table 5. Top 30 enriched proteins in immunoprecipitants of PA2G4 detected by MS.**

| **Gene names** | **Peptides** | **Unique peptides** | **Score** |
| --- | --- | --- | --- |
| KRT1 | 28 | 21 | 323.31 |
| KRT2 | 22 | 13 | 323.31 |
| VKA1 | 2 | 2 | 245.13 |
| H2AFV | 2 | 2 | 211.67 |
| RBM14 | 12 | 11 | 176.89 |
| KRT6B | 15 | 0 | 173.53 |
| KRT5 | 16 | 7 | 118.63 |
| RPL28 | 7 | 7 | 102.59 |
| RPL7 | 1 | 1 | 93.603 |
| HNRPH3 | 2 | 2 | 87.051 |
| **YTHDF2** | **3** | **3** | **86.662** |
| HIST1H4H | 2 | 2 | 80.255 |
| HRNR | 3 | 3 | 78.383 |
| RPS10 | 2 | 2 | 67.675 |
| HNRPU | 9 | 9 | 65.23 |
| RPS11 | 6 | 6 | 62.843 |
| RPL27 | 2 | 2 | 58.45 |
| ILF3 | 5 | 5 | 52.364 |
| H3F3B | 3 | 3 | 52.227 |
| ALB | 5 | 5 | 51.466 |
| RPS23 | 1 | 1 | 44.615 |
| HNRNPM | 7 | 7 | 41.507 |
| RPS4X | 6 | 6 | 36.614 |
| RPL26 | 4 | 4 | 24.344 |
| RPL19 | 1 | 1 | 23.278 |
| RPS14 | 2 | 2 | 22.777 |
| PABPC1 | 3 | 2 | 16.815 |
| HNRNPD | 1 | 1 | 16.074 |
| HNRNPUL1 | 10 | 10 | 14.725 |
| MATR3 | 5 | 5 | 11.941 |

**Supplementary table 6. Clinicopathologic characteristics of patients with hepatocellular carcinoma in cohort 3.**

| **Clinicopathological variables** | **Number**  **n=120** | **Percentage (%)** |
| --- | --- | --- |
| **Gender** |  |  |
| Male | 101 | 84.17 |
| Female | 19 | 15.83 |
| **Age** |  |  |
| ≤50 | 57 | 47.5 |
| >50 | 63 | 52.2 |
| **AFP (ug/L)** |  |  |
| ≤20 | 32 | 26.67 |
| >20 | 88 | 73.33 |
| **ALT (U/L)** |  |  |
| ≤41 | 87 | 72.5 |
| >41 | 33 | 27.5 |
| **AST (U/L)** |  |  |
| ≤40 | 88 | 73.33 |
| >40 | 32 | 26.67 |
| **GGT (U/L)** |  |  |
| ≤71 | 75 | 62.5 |
| >71 | 45 | 37.5 |
| **ALP (U/L)** |  |  |
| ≤130 | 104 | 86.67 |
| >130 | 16 | 13.33 |
| **HBV** |  |  |
| Negative | 0 | 0 |
| Positive | 120 | 100 |
| **HCV** |  |  |
| Negative | 120 | 100 |
| Positive | 0 | 0 |
| **Cirrhosis** |  |  |
| No | 33 | 27.5 |
| Yes | 87 | 72.5 |
| **Ascites** |  |  |
| No | 101 | 84.17 |
| Yes | 19 | 15.83 |
| **Tumor size** |  |  |
| ≤5 | 50 | 41.67 |
| >5 | 70 | 58.33 |
| **Tumor number** |  |  |
| Single | 93 | 77.5 |
| Multiple | 27 | 22.5 |
| **Tumor encapsulation** |  |  |
| None | 54 | 45 |
| Complete | 66 | 55 |
| **Macrovascular invasion** |  |  |
| No | 103 | 85.83 |
| Yes | 17 | 14.17 |
| **Microvascular invasion** |  |  |
| No | 108 | 90 |
| Yes | 12 | 10 |
| **Satellite nodules** |  |  |
| No | 92 | 76.67 |
| Yes | 18 | 23.33 |
| **Extrahepatic metastasis** |  |  |
| Negative | 119 | 99.17 |
| Positive | 1 | 0.83 |
| **Child-Pugh** |  |  |
| A | 113 | 94.16 |
| B | 7 | 5.84 |
| **BCLC stage** |  |  |
| 0+A | 72 | 60 |
| B+C | 48 | 40 |

**Supplementary table 7. Correlation between relative FYN expression and clinicopathologic characteristics of HCC patients in cohort 3 (n= 120).**

| **Clinicopathological variables** | **Relative FYN expression** | | **P value** | |  |
| --- | --- | --- | --- | --- | --- |
|  | **Negative**  **(n= 66)** | **Positive**  **(n=54)** |  |  |  |
| **Gender** |  |  |  | |  |
| Male | 53 | 48 | 0.199 | |  |
| Female | 13 | 6 |  | |  |
| **Age** |  |  |  | |  |
| ≤50 | 36 | 21 | 0.088 | |  |
| >50 | 30 | 33 |  | |  |
| **AFP (ug/L)** |  |  |  | |  |
| ≤20 | 14 | 18 | 0.135 | |  |
| >20 | 52 | 36 |  | |  |
| **ALT (U/L)** |  |  |  | |  |
| ≤41 | 48 | 39 | 0.951 | |  |
| >41 | 18 | 15 |  | |  |
| **AST (U/L)** |  |  |  | |  |
| ≤40 | 47 | 41 | 0.561 | |  |
| >40 | 19 | 13 |  | |  |
| **GGT (U/L)** |  |  |  | |  |
| ≤71 | 40 | 35 | 0.636 | |  |
| >71 | 26 | 19 |  | |  |
| **ALP (U/L)** |  |  |  | |  |
| ≤130 | 57 | 47 | 0.914 | |  |
| >130 | 9 | 7 |  | |  |
| **HBV** |  |  |  | |  |
| Negative | 0 | 0 | 1.000* | |  |
| Positive | 66 | 54 |  | |  |
| **HCV** |  |  |  | |  |
| Negative | 66 | 54 | 1.000* | |  |
| Positive | 0 | 0 |  | |  |
| **Cirrhosis** |  |  |  | |  |
| No | 21 | 12 | 0.242 | |  |
| Yes | 45 | 42 |  | |  |
| **Ascites** |  |  |  | |  |
| No | 61 | 40 | **0.006** | |  |
| Yes | 5 | 14 |  | |  |
| **Tumor size** |  |  |  | |  |
| ≤5 | 31 | 19 | 0.193 | |  |
| >5 | 35 | 35 |  | |  |
| **Tumor number** |  |  |  | |  |
| Single | 52 | 41 | 0.709 | |  |
| Multiple | 14 | 13 |  | |  |
| **Tumor encapsulation** |  |  |  | |  |
| None | 33 | 21 | 0.224 | |  |
| Complete | 33 | 33 |  | |  |
| **Macrovascular invasion** |  |  |  | |  |
| No | 56 | 47 | 0.732 | |  |
| Yes | 10 | 7 |  | |  |
| **Microvascular invasion** |  |  |  | |  |
| No | 60 | 38 | **0.038** | |  |
| Yes | 6 | 16 |  | |  |
| **Satellite nodules** |  |  |  | |  |
| No | 53 | 39 | 0.298 | |  |
| Yes | 13 | 15 |  | |  |
| **Extrahepatic metastasis** |  |  | |  | |
| Negative | 65 | 54 | 1.000* | |  |
| Positive | 1 | 0 |  | |  |
| **Child-Pugh** |  |  |  | |  |
| A | 65 | 48 | **0.026*** | |  |
| B | 1 | 6 |  | |  |
| **BCLC stage** |  |  |  | |  |
| 0+A | 45 | 27 | **0.043** | |  |
| B+C | 21 | 27 |  | |  |

Chi-square test was used to analyze categorical data; *, Fisher exact test.

**Supplementary table 8. Primers for qRT-PCR analysis.**

| **Gene name** | **Forward primer** | **Reverse primer** |
| --- | --- | --- |
| YTHDF2 | AGCCCCACTTCCTACCAGATG | TGAGAACTGTTATTTCCCCATGC |
| FYN | ATGGGCTGTGTGCAATGTAAG | GAAGCTGGGGTAGTGCTGAG |
| PA2G4 | GACATCGCCAACAGGGTACTT | TTACCGAAATGCTGGTGGGA |
| GAPDH | CTGGGCTACACTGAGCACC | AAGTGGTCGTTGAGGGCAATG |
| PNMA2 | ACCACCAGCTAATGGATGCG | CCCTGTTGATGTGTTCCCGA |
| ICA1 | CTTCGATCCCAAGGTTTCCAA | TCGACACAAAGGATTTCGTAAGG |
| OLR1 | TTGCCTGGGATTAGTAGTGACC | GCTTGCTCTTGTGTTAGGAGGT |
| HPRT1 | TGACACTGGCAAAACAATGCA | GGTCCTTTTCACCAGCAAGCT |

**Supplementary materials and methods:**

**Western blot**

Western blot analysis and quantification of optical densities were performed as described previously(3). Primary antibody used for western blot analyses included anti-PA2G4 (66055-1-Ig, Proteintech), anti-GAPDH (60004-1-Ig, Proteintech), anti-FYN (4023S, CST), anti-E-cadherin (610182, BD), anti-N-cadherin (610921, BD), anti-ZEB1 (21544-1-AP, Proteintech), anti-ZEB2 (14026-1-AP, Proteintech), anti-Vimentin (#5741, CST), anti-Occludin (27260-1-AP, Proteintech) and anti-YTHDF2 (24744-1-AP, Proteintech). Horseradish Peroxidase (HRP) conjugated goat anti-rabbit IgG and goat anti-mouse IgG were purchased from Promoter (Wuhan, China).

**Immunohistochemistry (IHC):**

IHC analyses were performed as previously described(3) to determine *in suit* expression level of PA2G4 and FYN in HCC and normal tissues. Anti-PA2G4 (66055-1-Ig, Proteintech), anti-FYN (4023S, CST), universal SP kit (SP-9000, ZSGB-BIO) and DAB substrate (ZLI-9019, ZSGB-BIO) were used for IHC procedure according to manufactures’ protocol.

**Transwell assay**

8μm pore size 24-well-transwell plates (Corning, NY, USA) were used to evaluated cell mobility of HCC cells as previously described(3). 5x10^4^/10x10^4^ Huh7, 1x10^4^/3x10^4^ HLF and 10x10^4^/20x10^4^ HCC-LM3 cells per chamber were used for migration/invasion assay. 24 hours after cells seeding, the chambers were collected for crystal violet staining. Three random views were captured under 100x magnification by microscope (Leica, Solms, Germany) for each chamber. The number of staining cells were quantified by Image-Pro Plus v6.0 software.

**Generation of lentivirus for PA2G4 overexpression and knockdown:**

Three independent shRNAs targeting PA2G4 were designed by GPP Web Portal, and cloned to the plko.1-TRC vector (10879, Addgene, Cambridge, MA). Non-targeting scramble sequence was used as control. pMD2G and pxPax2 were used for lentivirus packaging plasmids. The lentivirus packaging procedures were performed as previously described(3). The sequence of shRNA and scramble oligos were listed below.

scramble: CCTAAGGTTAAGTCGCCCTCG

shPA2G4-1#: CCTGGTCGTGACCAAGTATAA

shPA2G4-2#: CGCTAATGTAGCTCACACTTT

shPA2G4-3#: GCCGTTTACTTTAAGAGCATT

Lentivirus for stably overexpressing PA2G4 was purchased from DesignGene Biotechnology (Shanghai, China). rLV-ZsGreen-Puro vector was used as backbone for generating PA2G4 overexpressing lentivirus.

**RNA isolation and qRT-PCR analysis**

Total RNA was extracted from cells using RNAiso Plus reagent (9108/9109, Takara) as previously described. HiScript® II Q RT SuperMix (R223, Vazyme) was used for reverse transcribing cDNA. qRT-PCR was performed using ChamQ Universal SYBR qPCR Master Mix (Q711, Vazyme) according to manufacturers’ introductions. GAPDH was used as internal controls. Comparative CT (2^−∆∆CT^) method was used to perform relative quantification analysis and each experiment was independently repeated three times at least. Primers for qRT-PCR analysis were listed in supplementary table 8.

**RNA-Sequencing (RNA-Seq):**

RNA-Seq experiment and high through-put sequencing and data analysis were conducted by Seqhealth Technology Co., LTD (Wuhan, China). Briefly, total RNAs were extracted from HCC cells using TRIzol Reagent (NO.15596026, Invitrogen). DNA digestion was carried out after RNA extraction by DNaseI. 2 μg total RNAs were used for stranded RNA sequencing library preparation using KC^TM^ Stranded mRNA Library Prep Kit for Illumina® (Catalog NO. DR08402, Wuhan Seqhealth Co., Ltd. China) following the manufacturer’s instruction. PCR products corresponding to 200-500 bps were enriched, quantified and finally sequenced on Novaseq 6000 sequencer (Illumina) with PE150 model. Raw sequencing data was first filtered by Trimmomatic (version 0.36), low-quality reads were discarded and the reads contaminated with adaptor sequences were trimmed. Clean data were mapped to the reference genome of Homo sapiens from GRCh38 using STRA software (version 2.5.3a) with default parameters. Reads mapped to the exon regions of each gene were counted by featureCounts (Subread-1.5.1; Bioconductor) and then RPKMs were calculated. Genes differentially expressed between groups were identified using the edgeR package (version 3.12.1). A p-value cutoff of 0.05 and fold-change cutoff of 2 (log2(FC)≥1 and p value<0.05) were used to judge the statistical significance of gene expression differences. Gene ontology (GO) analysis and Kyoto encyclopedia of genes and genomes (KEGG) enrichment analysis for differentially expressed genes were both implemented by KOBAS software (version: 2.1.1) with a p-value cutoff of 0.05 to judge statistically significant enrichment.

**Regents, siRNA and plasmids:**

N-(3,4,-Dimethoxycinnamoyl) anthranilic acid (DAA) (B7827, ApexBio) was purchased from Promoter, Wuhan. Small interference RNAs (siRNA) targeting FYN were purchased from Ribobio (Guangzhou, China). The genomic sequence located between −200 and +1000 base pairs (bp) from the transcription start site (TSS) of FYN was cloned into PGL4.17 [luc2/Neo] vector (E672A, Promega) for dual luciferase reporter assay. The coding sequence of PA2G4, FYN or YTHDF2 was cloned into the pcDNA3.1+ vector (Addgene, 02139) for transient overexpression. For the transient overexpression of non-catalytic YTHDF2 (YTHDF2-5A), the K416/R527/W432/W486/W491 on the CDS of YTHDF2 were mutated to alanine(4).

Lipofectamine 3000 transfection reagent (Thermo Fisher Scientific, Waltham, MA, United States) was used to transfected plasmids or siRNAs into cells according to the manufacturer’s instructions. The sequences of siRNA targeting FYN were listed below.

negative control (nc): TTCTCCGAACGTGTCACGT

siFYN-1#: CGCATGAATTATATCCATA,

siFYN-2#:CAACTGGAGAGACAGGTTA,

siFYN-3#: GGAGACCATGTCAAACATT

**Immunoprecipitation assay (IP):**

Endogenous IP assay was performed as previously described(3). Anti-PA2G4 (15348-1-AP, Proteintech; 1:100) and anti-YTHDF2 (24744-1-AP, Proteintech; 1:150) were used to precipitate their respective binding proteins. Normal Rabbit IgG (#2729, Cell signaling technology; 1:500) was used as negative control. Anti-PA2G4 (15348-1-AP, Proteintech) and anti-YTHDF2 (24744-1-AP, Proteintech) were applied as the primary antibody for western blot analysis of IP samples. IPKine™ HRP, Goat Anti-Rabbit IgG HCS (A25222, Abbkine) and IPKine™ HRP, Goat Anti-Mouse IgG HCS (A25112, Abbkine) were used as secondary antibody to eliminated the interference of light chain.

**Sliver staining and** **Liquid chromatography tandem-mass spectrometry (LC-MS):**

HEK-293T cells were transduced with pcDNA3.1-PA2G4 to ectopically overexpress PA2G4. Anti-PA2G4 precipitates in HEK-293T cells were separated by SDS-PAGE gel and stained with Fast Silver Stain Kit (P0017S, Beyotime, China). LC-MS/MS was performed and analyzed by Shanghai Bioprofile Technology Co., Ltd. (China).

**Immunofluorescence and confocal assay:**

Cells were seeded on sterile coverslips and cultured overnight. 4% paraformaldehyde was used for fixation, and followed by 0.5% Triton X-100 for permeabilization at room temperature. Primary antibodies (anti-PA2G4: anti-YTHDF2) were mixed at 1:1 ratio for antibody cocktails (final concentration: 2 μg/ml). Antibody cocktails were added to cells to incubated overnight at 4°C, and washed with PBST for 6 times before subjected to secondary antibodies. Cy3-conjucated AffiniPure Goat Anti-Rabbit IgG (111-165-003, Jackson ImmunoResearch Laboratory) and FITC-conjugated AffiniPure Goat Anti-Mouse IgG (111-095-003, Jackson ImmunoResearch Laboratory) were used as secondary antibodies to incubate cell samples for 1 hours. After washed with PBST for 6 times, DAPI was used to counterstain cell nuclei. F-actin stress fibers were stained with Phalloidin-iFluor™ 594 Conjugate (23122, AAT Bioquest). Images were taken by confocal laser-scanning microscopy (Zeiss LSM 800).

**References:**

1. Ding ZY, Jin GN, Wang W, Chen WX, Wu YH, Ai X, et al. Reduced Expression of Transcriptional Intermediary Factor 1 Gamma Promotes Metastasis and Indicates Poor Prognosis of Hepatocellular Carcinoma. Hepatology. 2014;60(5):1620-36.

2. Giannelli G, Fransvea E, Marinosci F, Bergamini C, Colucci S, Schiraldi O, et al. Transforming growth factor-beta1 triggers hepatocellular carcinoma invasiveness via alpha3beta1 integrin. Am J Pathol. 2002;161(1):183-93.

3. Huang Z, Chu L, Liang J, Tan X, Wang Y, Wen J, et al. H19 Promotes HCC Bone Metastasis Through Reducing Osteoprotegerin Expression in a Protein Phosphatase 1 Catalytic Subunit Alpha/p38 Mitogen-Activated Protein Kinase-Dependent Manner and Sponging microRNA 200b-3p. Hepatology. 2021;74(1):214-32.

4. Zhong L, Liao D, Zhang M, Zeng C, Li X, Zhang R, et al. YTHDF2 suppresses cell proliferation and growth via destabilizing the EGFR mRNA in hepatocellular carcinoma. Cancer Lett. 2019;442:252-61.
